# Self and parasite-derived peptides selected upon DERAA-bearing HLA-DRB1 alleles activate CD4+ T cells from Chagas cardiomyopathy patients and are associated with ventricular dysfunction

**DOI:** 10.3389/fimmu.2025.1527115

**Published:** 2025-05-05

**Authors:** Thaiany G. Souza-Silva, Eula G. A. Neves, Andrea Teixeira-Carvalho, Amanda Braga Figueiredo, Katia Luciano Pereira Morais, Juliana Apostólico, Hélcio Rodrigues, Jorge Kalil, Maria Aparecida Juliano, Luiz Juliano, Silvana Silva Araújo, Alexandre Negrão Pantaleao, Antônio Mutarelli, Maria Carmo Pereira Nunes, Kenneth J. Gollob, Walderez O. Dutra

**Affiliations:** ^1^ Laboratório Biologia das Interações Celulares, Depto. Morfologia, Instituto de Ciências Biológicas, Belo Horizonte, MG, Brazil; ^2^ Instituto René Rachou, Fundação Oswaldo Cruz (FIOCRUZ), Belo Horizonte, MG, Brazil; ^3^ Center for Research in Immuno-Oncology (CRIO), Hospital Israelita Albert Einstein, São Paulo, SP, Brazil; ^4^ Laboratory of Immunology, Heart Institute, Instituto do Coração (InCor), School of Medicine, University of São Paulo, São Paulo, SP, Brazil; ^5^ Departamento de Biofísica, Escola Paulista de Medicina, UNIFESP, São Paulo, SP, Brazil; ^6^ Depto. Clínica Médica, Faculdade de Medicina, Universidade Federal de Minas Gerais, Belo Horizonte, MG, Brazil; ^7^ Instituto Nacional de Ciência e Tecnologia em Doenças Tropicais, Instituto Nacional de Ciência e Tecnologia em Doenças Tropicais (INCT-DT), Belo Horizonte, Brazil

**Keywords:** Chagas disease, HLA-DRB1, cardiomyopathy, cross-reactivity, T-cells

## Abstract

**Introduction:**

Human infection with the protozoan *Trypanosoma cruzi* causes Chagas disease, which may lead to a deadly dilated cardiomyopathy resulting from T-cell-mediated inflammation. We found that specific HLA-DRB1 alleles (*0103, *0402, *1301, and *1302) that display the DERAA motif are linked to this severe clinical manifestation of Chagas disease.

**Methods:**

We employed computational analysis, *in vitro* functional assays, and single-cell RNA sequencing to determine the response of CD4+ T cells from indeterminate (IND) and cardiac (CCC) Chagas patients to peptides selected on DERAA-bearing alleles.

**Results:**

We observed that these alleles display binding affinity towards host-derived peptides with sequence similarity to parasite-derived proteins. These peptides can activate and induce proliferation of CD4+ T-cells from CCC, but not IND. Importantly, the magnitude of this response correlated with the severity of ventricular dysfunction and increased production of soluble factors associated with myocardial fibrosis. Analysis of differentially expressed genes (DEGs) in activated CD4+ T-cells from individuals with the DERAA-DRB1 alleles demonstrated a high expression of cytotoxic, chemotactic and proapoptotic genes, linking these cells with pathogenic functions. Finally, we observed the upregulation of genes that code for the host proteins that contain the potentially pathogenic peptides in the cardiac tissue of CCC, suggesting their involvement in cardiomyopathy.

**Discussion:**

Our findings highlight the ability of CD4+ T-cells from CCC patients to recognize and react to foreign and self-peptides, thereby emphasizing the importance of HLA-DRB1 alleles in the presentation of potentially pathogenic antigens and in the amplification of CCC pathology.

## Graphical Abstract

CD4+ T cells from Chagas disease patients carrying DERAA-DRB1 alleles show increased expression of cytotoxic, chemotactic, and proapoptotic genes, whereas patients carrying non- DERAA-DRB1 alleles show upregulation of survival, antiapoptotic and anergy-related genes. CD4+T cells from DERAA donors recognize and bind to host-derived peptides with sequence similarity to parasite proteins, leading to CD4+ T cell activation and proliferation, a response not observed in non-DERAA donors.

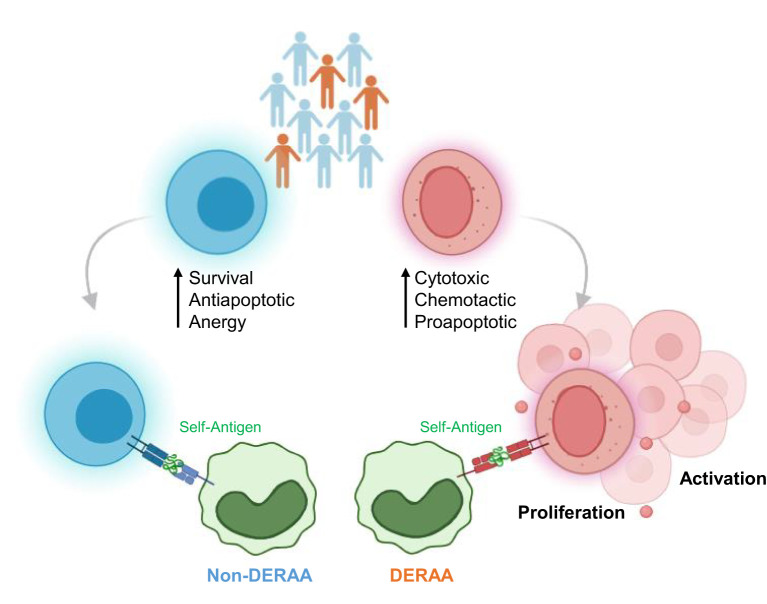

## 1 Introduction

The Human Leukocyte Antigen (HLA) and Major Histocompatibility Complex (MHC) loci represent highly polymorphic regions within the human genome (1, 2). This characteristic is crucial for generating allelic diversity, facilitating the recognition and presentation of diverse pathogen-derived antigens. HLA genes are situated on chromosome six and are categorized into specific regions for classes I, II, and III, which participate not only in peptide presentation to T lymphocytes, but also in encoding cytokines and proteins of the complement system (1, 2). The HLA-II region comprises three loci housing the HLA-DR, HLA-DQ, and HLA-DP genes. Proteins derived from HLA are indispensable for T-cell activation and the establishment of effective adaptive immune responses during pathogen infections, including parasites such as *Toxoplasma gondii* (3), *Plasmodium* (4), *Leishmania major* (5), and *Trypanosoma cruzi* (6, 7). These interactions play a pivotal role in evoking protective and pathogenic immune responses.

Human infection with *T. cruzi* leads to Chagas disease, a debilitating illness affecting approximately 6 million individuals and with millions at risk (8). While around 60-70% of chronically infected patients present with no evidence of organ involvement, falling under the indeterminate clinical form (IND), 30% progress to a debilitating and life-threatening cardiomyopathy, characterizing chronic Chagas cardiomyopathy (CCC) (9, 10). CCC results from a robust inflammatory response involving activation of T-cells by both parasite and host antigens (11–15), suggesting a role for cross-reactive responses in disease pathogenesis.

Numerous studies have reported a link between specific HLA-DRB1 alleles and an increased susceptibility to severe forms of infectious diseases (16–18). We previously demonstrated that more than 60% of CCC patients express at least one of the HLA-DRB1 alleles (*0103, *0402, *1301, and *1302) which harbor the motif containing aspartic acid - glutamic acid – arginine – alanine - alanine (DERAA) (7). The DERAA motif is a conserved sequence within the beta chain of certain HLA-DR molecules (19, 20), that has been associated with diseases. DRB1 allele groups encoding DERAA have been linked to protection in autoimmune diseases, such as rheumatoid arthritis, systemic lupus erythematosus, psoriasis, and systemic sclerosis (21, 22). However, in Chagas and Whipple’s diseases, caused by infection with a protozoan and a bacterium, respectively, the HLA-DRB1 bearing the DERAA motif has been associated with disease severity (7, 18). Identifying parasite and host epitopes that interact with these specific HLA-DRB1 molecules is key to revealing antigens that contribute to disease pathogenesis.

In this study we found that DERAA-bearing HLA-DRB1 alleles exhibited high affinities for host-derived peptides, despite their inability to directly recognize parasite-derived peptides based on *in silico* binding predictions. Notably, these host-derived peptides presented significant similarity to sequences found in parasite-derived proteins. Furthermore, we showed that CD4+ T-cells from CCC patients, but not IND, can recognize and mount immune responses against both foreign and self-peptides selected upon DERAA-bearing HLA-DRB1 alleles. This response is characterized by the expression of genes associated with proapoptotic and cytotoxic profiles in patients that carry the DERAA allele. Importantly, the intensity of this response correlates with clinical indicators of impaired ventricular function, providing compelling evidence as to the involvement of HLA-DRB1 alleles in the selection of pathogenic antigens, promotion, and amplification of cardiac pathology in Chagas disease.

## 2 Material and methods

### 2.1 Epitope mapping for HLA-DRB1 alleles

The human and *T. cruzi* epitopes restricted to HLA-DRB1 *0103, *0402, *1301, and *1302 alleles, as well as T and B cell immunogens, were identified using the Immune Epitope Database (IEDB) (www.iedb.org) (23). The selection of these specific HLA-DRB1 alleles was based on the study conducted by Menezes et al. (2012), which demonstrated a high prevalence of these alleles in patients with CCC. To analyze the amino acid sequences of all peptides for similarity to *T. cruzi* (Taxid:5693) proteins, the Basic Local Alignment Search Tool (BLAST) from the National Center for Biotechnology Information (NCBI) was employed (24). Protein sequences were obtained from UniProt, and regions exhibiting similar amino acid sequences between human peptides and *T. cruzi* peptides were utilized to predict T and B cell epitopes. For subsequent analyses, only epitopes containing a minimum of 9 amino acids (9-mer) were considered.

### 2.2 Binding affinity HLA-DRB1-peptide

Binding affinity of HLA-DRB1-peptide interactions was evaluated for all selected epitopes (both human and *T. cruzi* peptides) using the NetMHCIIpan tool (version 4.0) (25). Peptides with a binding affinity below the threshold of 100nM (Aff) were classified as strong binders, while those with a threshold greater than 1000nM were classified as weak binders. Peptides falling within the range of 100 to 1000nM were categorized as intermediate binders (26). Additionally, the binding core of all peptides was predicted using the NetMHCIIpan method version 4, accessible at http://www.cbs.dtu.dk/services/NetMHCIIpan/.

### 2.3 Anchor-motif and peptide-HLA-DR structural analysis

We employed the Peptide Repertoire-Based Anchor Motif software (PRBAM) to predict the interactions between peptides and HLA-II *0103, *0402, *1301, and *1302 alleles and to identify the anchor residues of amino acids (27). We performed protein-peptide docking using crystal structures of HLA-DRB1 molecules retrieved from the Protein Data Bank (PDB). Specifically, the crystal structures of HLA-DRB10103 (PDB ID: 3PDO_B), HLA-DRB10402 (PDB ID: 1J8H_B), and HLA-DRB1*13 allele (PDB ID: 1AQD_B) obtained from the PDB (http://www.rcsb.org/pdb/) were utilized for the docking process. The docking was performed using the HPEPDOCK server (http://huanglab.phys.hust.edu.cn/hpepdock/), an online tool that employs a fast rigid-body peptide-protein docking algorithm based on a hierarchical approach to predict interactions between proteins and ligands (28). The resulting peptide-HLA-II interactions were visualized using PyMOL software, and the anchor positions of amino acid residues at positions P1, P4, P6, and P9 in the HLA-DR binding groove were analyzed using PyMOL software (The PyMOL Molecular Graphics System, Version 1.2r3pre, Schrodinger, LLC) (26).

### 2.4 Prediction of immunogenicity and antigenicity and of cytokine secretion profile

For predicting the capacity of human peptides to elicit an immune response, we utilized the CD4+ T Cell Immunogenicity prediction tool (http://tools.iedb.org/CD4episcore) from the IEDB. This analysis considered allele-independent CD4+ T cell immunogenicity and 7-allele HLA binding (DRB1 *03:01, *07:01, *1501, DRB3 *01:01, *02:02, DRB4 *01:01, DRB5 *0101) (29, 30). Additionally, we predicted the immunogenicity of the corresponding *T. cruzi* peptides. Peptides with an immunogenicity score below 73 were identified as more immunogenic, while peptides with scores ranging from 73 to 99 were considered non-immunogenic (29–31).

The IFNepitope server (https://webs.iiitd.edu.in/raghava/ifnepitope/application.php) was utilized to assess the ability of peptides to induce IFN-γ secretion by CD4+ T-cells. Peptides with a threshold of 0.0 were classified as IFN-γ inducers, while those with negative results were categorized as non-inducers (32). To evaluate the potential of the peptides to induce IL-4, the IL4Pred web server was employed (http://crdd.osdd.net/raghava/il4pred/index.php). This server distinguishes IL-4 inducers (threshold = 0.2) from non-inducers (threshold < 0.2) based on the pattern of the input sequences (33).

### 2.5 *In silico* analysis of differently expressed genes in cardiac tissue

The dataset containing the transcript expression profile of human left ventricular lateral wall heart tissues from CCC donors (10 advanced heart failure patients) and healthy donors (7 healthy heart tissues) was obtained from the study by Laugier et al. (34). The gene expression data files are accessible under the accession number GSE84796 of the Gene Expression Omnibus. GEO2R (http://www.ncbi.nlm.nih.gov/geo/geo2r/) was employed to identify differentially expressed mRNAs between healthy donors and CCC heart tissue samples. Subsequently, the expression levels of all human peptides identified in the IEDB database as ligands for the HLA-DR *0103, *0402, *1301, and *1302 alleles were evaluated in CCC and healthy donor heart tissues. GEO2R, with an interface similar to R-packages, facilitated the evaluation, identification, and visualization of GEO data (35). The correction of false-positive *P*-value results was carried out using the Benjamin and Hochberg false discovery rate method.

### 2.6 Patients enrolled

For this cross-sectional study, 10 volunteer patients, carefully classified into the polar forms of Chagas disease (IND n=5 and CCC n=5), were recruited from tertiary center for Chagas disease at the Federal University of Minas Gerais (UFMG) who were referred for the management of Chagas disease. All patients tested positive for *T. cruzi* serology, as confirmed by ELISA, hemagglutination, and immunofluorescence assays. The patients were submitted to a comprehensive clinical assessment, allowing for their unequivocal classification into the distinct and polar clinical forms of Chagas disease, as described below. The parameters of proliferation, cytokine expression and single-cell RNA sequencing (scRNAseq) were evaluated for all the selected patients who followed the clinical criteria, providing a comprehensive multiparametric analysis of their immune profile.

The clinical forms of Chagas disease were determined using several exams, including: clinical history assessment to evaluate symptoms and functional class, physical examinations, electrocardiogram (ECG), chest X-rays, and echocardiogram, in accordance with previously established criteria (36). The conjunction of the results allowed for the refined clinical characterization of the patients. Asymptomatic Chagas patients with positive serology against *T. cruzi* but without any clinical manifestations or cardiac alterations related to Chagas disease were classified into the indeterminate clinical form (IND). Patients classified as having chronic Chagas cardiomyopathy (CCC) displayed ECG abnormalities and/or left ventricular dilation with systolic dysfunction (36). Echocardiographic data, including the left ventricular ejection fraction (LVEF), left ventricular end-systolic diameter (LVSD), left ventricular end-diastolic diameter (LVDD), and HLA-DRB1 typing were measured and are presented in **Table 1**. Quantification of left ventricular diameters and function were obtained in accordance to the recommendations of the American Society of Echocardiography guidelines (37). LVDD was measured at the end-diastole in the long-axis view and LVEF was derived by calculating the variance between end-diastolic and end-systolic volumes and then dividing it by the “end-diastolic volume”. Exclusion criteria included: diabetes mellitus, thyroid dysfunction, renal insufficiency, chronic obstructive pulmonary disease, rheumatic heart disease, as well as any autoimmune disease or chronic inflammatory disorders. This study was approved by the Ethics Committee on Research of the Federal University of Minas Gerais (COEP-UFMG-ETIC077/06), and all participants provided informed consent and were aware of the research purpose.

**Table 1:** Clinical and demographic features of Chagas disease patients.

Patients	Clinical form	Sex	HLA-DRB1		Age (Years)	LVEF (%)	LVDD (mm)	LVSD (mm)
			Allele 1	Allele2				
D1	IND	F	*1601	*1601	55	68	48	30
D2	IND	M	*1301	*1503	64	68	48	31
D3	IND	F	*0301	*0701	62	71	47	28
D4	IND	M	*0701	*1602	67	68	49	33
D5	IND	M	*0403	*0405	63	67	48	30
D6	CCC	F	*0701	*1301	65	45	68	53
D7	CCC	M	*1001	*1601	59	50	49	36
D8	CCC	F	*1301	*1501	67	54	64	55
D9	CCC	F	*0101	**1503	56	63	55	32
D10	CCC	F	*0411	*0801	58	48	43	58
p value (IND vs CCC)					0.69	0007**	0.18	0.03**

IND, Indeterminate; CCC, chronic Chagas cardiomyopathy; LVEF, left ventricular ejection fraction; LVDD, left ventricular end-diastolic diameter; LVSD, left ventricular end-systolic diameter.

*Bold type: alleles related to the DERAA motif. Clinical forms of Chagas disease: IND, Indeterminate; CCC, Chagas cardiomyopathy.

**p-value indicates statistically significant differences between IND and CCC groups.

### 2.7 Peptide synthesis

Peptides were selected for synthesis based on the following criteria: (i) strong or intermediate binding affinity to HLA-DRB1 alleles, (ii) better docking score, (iii) potential immunogenicity. Peptides were synthesized using an automated multiple peptide synthesizer (PSSM-8 system Shimadzu, Tokyo, Japan) following a previously described methodology (38). The peptide synthesis employed the NovaSyn*TGR resin (Millipore, USA) with HBTU (N,N’-tetramethyl-O-benzotriazo-1-yluronium tetrafluoroborate)/HOBt (1-hydroxybenzotriazole) as the coupling reagent. Cleavage of peptides from the resin was achieved using TFA:thioanisole:1,2-ethanedithiol:water (85:5:3:7) mixture. Purification was carried out using semi-preparative HPLC on a C18 column (EconosilTM; Fisher Scientific, USA), and the molecular mass was determined using reverse-phase chromatography and mass spectrometry (38, 39). The peptides (purity 94-96%) were dissolved in DMSO, diluted to a concentration of 1 mg/ml, and stored at -20 °C until further use.

### 2.8 Blood collection, peripheral blood mononuclear cells purification and *in vitro* stimulation

Peripheral blood samples were collected from all volunteers via venipuncture using vacutainer tubes with anticoagulant (heparin or EDTA, Vacutainer, Becton Dickinson, San Jose, CA, USA). Peripheral blood mononuclear cells (PBMCs) were isolated by layering whole blood over Ficoll-Paque PLUS (G.E Healthcare, Sweden) following the protocol described by Souza et al. (40). The resulting leukocytes were washed three times with phosphate-buffered saline (PBS 1x) and labeled with CFSE (carboxyfluorescein diacetate succinimidyl ester-Molecular Probes C1157) using a previously published method with modifications (41). In brief, 4.5 x 10^6^ PBMCs were incubated with 0.1 mM CFSE for 15 minutes at 37 °C and 5% CO2. The labeled PBMCs were then washed three times with cold PBS + 10% inactivated fetal calf serum (FCS) by centrifugation at 600 x g for 10 minutes at 4°C.

CFSE-labeled PBMCs were resuspended in RPMI 1640 medium (Thermo Fisher Scientific, Waltham, US), supplemented with 5% inactivated human serum (Thermo Fisher Scientific, Waltham, US), 1% antibiotic (penicillin, 200 U/mL; and streptomycin, 0.1 mg/mL, Thermo Fisher Scientific, Waltham, US), and 1 mM L-glutamine (Sigma-Aldrich, St. Louis, US) at a concentration of 1 x 10^7^ cells/mL. CFSE-labeled PBMCs (3 x 10^5^ cells/well) from each volunteer were stimulated under two conditions: (i) media plus anti-CD28 (0.5 µM/mL) and (ii) peptide (1 µg/mL of each peptide/donor) plus anti-CD28 (0.5 µM/mL) in a 96 U-bottom plate for 120 hours. The concentration of the peptide used to stimulate PBMCs was determined based on previous studies (42–44), and the stimulation time was determined through a pilot assay, which demonstrated that 120 hours, but not 72 hours, was the optimal protocol for measuring T lymphocyte proliferation (**Supplementary Table 1**).

### 2.9 Flow cytometry and cell sorting

CFSE-labeled PBMCs exposed to various stimuli were harvested after 120 hours of culture and subjected to specific staining using a mixture of monoclonal antibodies targeting surface molecules, conjugated to fluorochromes. The samples were incubated with the antibody mix, consisting of anti-CD4-Percp-Cy5 (clone A161A1), anti-CD8-APCcy7 (clone SK1), anti-CD19-PE (clone HIB19), and anti-HLA-DR-Pecy7 (clone L243), for 30 minutes at 4°C. Subsequently, the cells were washed with a cold solution of Wash B (PBS containing 0.5% BSA and 2 mM azide) and fixed using a 2% paraformaldehyde solution for 20 minutes. After fixation, the cells were washed with PBS and permeabilized for 15 minutes using 0.5% saponin. Permeabilized cells were then intracellularly stained with CD69-APC (clone FN50). The anti-CD19 antibody was obtained from BD Pharmingen (USA), while all other antibodies were sourced from Biolegend (San Diego, CA, USA).

The samples were acquired using the FACS CANTO II flow cytometer (Becton & Dickinson, San Jose, CA, USA) and analyzed using FlowJo software *version* 10.8.1_CL (Ashland, Oregon, USA), employing a supervised analysis strategy. The gating strategy involved removing doublet cells by analyzing forward scatter area (FSC-A) vs. forward scatter height (FSC-H). Live cells were selected based on Side Scatter Area (SSC-A) vs. BV421 fluorescence for the viability stain (Live-Dead). The lymphocyte population was identified by analyzing FSC-A vs. SSC-A, and different lymphocyte subpopulations were further analyzed as illustrated in **Supplementary Figure 1A**.

For cell sorting, PBMC were acquired as described above. PBMC were stained in sterile PBS with CD45 (Percp cy5.5) antibody, and DRAQ7 (APC-H7) and calcein (FITC) dyes before sorting on a BD FACS Aria FUSION ™ (SBIBAE-EC-003381) (**Supplementary Figure 1C**).

### 2.10 Single-cell RNA sequencing

CD45-positive cells were isolated and stained with oligonucleotide-conjugated Sample Tags following the manufacturer’s instruction using the BD™ Single-Cell Multiplexing Kit, BD-Biosciences. Single-cell capture and cDNA library preparation was performed using the BD Rhapsody Express Single-cell analysis system (BD Biosciences), using the BD Rhapsody cDNA (BD Biosciences, cat# 633663) and Target amplification (BD Biosciences, cat# 633664) kits, according to the manufacturer’s instructions. Sequencing was performed on Nova Seq™ 6000 (Illumina, San Diego, CA).

Data processing was initially conducted through the Seven Bridges Genomics platform, using BD Rhapsody Target Analysis Pipeline (version CWL-v1.2) to identifies and correct artifacts of sequencing, ensuring accurate molecule counting pre cell. DBEC (Distribution-based error correction) files were imported into SeqGeq version 1.6.0 (BD, Ashland, OR). Normalization was performed considering all genes, 10,000 counts per cell as a scaling factor. Quality control of samples was performed considering both cell and gene quality. Quality control for cell removes outlier events or doublets based on number of expressed genes relative to the library size. Gene quality control considered the total expression of each gene versus the cells expressing each gene, as well as the expression dispersion (**Supplementary Figure 1D**). The Lex BDSMK plugin was employed to identify Sample Tags. Data analyses were performed on sample-normalized and quality-controlled cells. Differentially expressed genes (DEGs) were identified using the FindMarker function, applying a Log2 fold change threshold (≥ 1.5 or ≤ -1.5) and a Bonferroni-adjusted *p* value<0.05. Our final dataset encompassed 61,938 total cells (29,094 CD4+ T cells) and 397 transcripts, providing a comprehensive analysis of genes expression patterns in the study. The graphs of gene expression were generated on SeqGeq and SRplot platform.

### 2.11 Analysis of soluble mediators

Plasma levels of soluble mediators were quantified using the Bio-plex ProTM Human Cytokine Assays 48-plex Kit (Bio-Rad Laboratories, Hercules, CA, USA) in accordance with the manufacturer’s instructions. Data acquisition was performed using a Bio-Plex 200 instrument, and the results were expressed as pg/mL, considering the standard curves provided in the kit and generated for each experimental batch.

### 2.12 DNA extraction and HLA typing

DNA was extracted from PBMCs using silica method (45, 46). PBMC pellets were washed twice with 450 µl of washing buffer (6.0 _M_ GuSCN, 65 m_M_ Tris-HCl pH 6.4), followed by two washes with 450 µl acetone, and then dried at 56°C for 20 min. Subsequently, 100 µl of TE buffer (10 m_M_ Tris-HCl pH 8.0 and 1m_M_ EDTA) was added, and the mixture was incubated at 56°C for 12h to release the DNA. After homogenization, the solution was centrifuged, and the supernatant containing DNA was collected (46). DNA quantification was performed using a Nanodrop spectrophotometer (NanoDrop Technologies, Inc, Wilmington, DE). HLA-II typing was conducted as previously described (47), using polymerase chain reaction and amplification using sequence-specific oligonucleotide contained in LABType kits (One Lambda Inc., Canoga Park, CA, USA).

### 2.13 Statistical analysis

The statistical analysis was conducted using Prism 8.0.2 (GraphPad Software, San Diego, CA, USA). The normality of the data was assessed using the Shapiro-Wilk test. Unpaired t-tests and Mann-Whitney tests were employed to compare parametric and non-parametric data, respectively, between the CCC and IND groups. The proliferation index was calculated by comparing stimulated and non-stimulated cells from the same donor using paired t-tests or Wilcoxon tests for parametric or non-parametric data, respectively. Correlation analyses were performed using Pearson or Spearman tests for parametric or non-parametric data, respectively. Statistical significance was defined as p < 0.05.

## 3 Results

### 3.1 DERAA-bearing HLA-DRB1 alleles have the potential to recognize self-peptides

**Figure 1:**
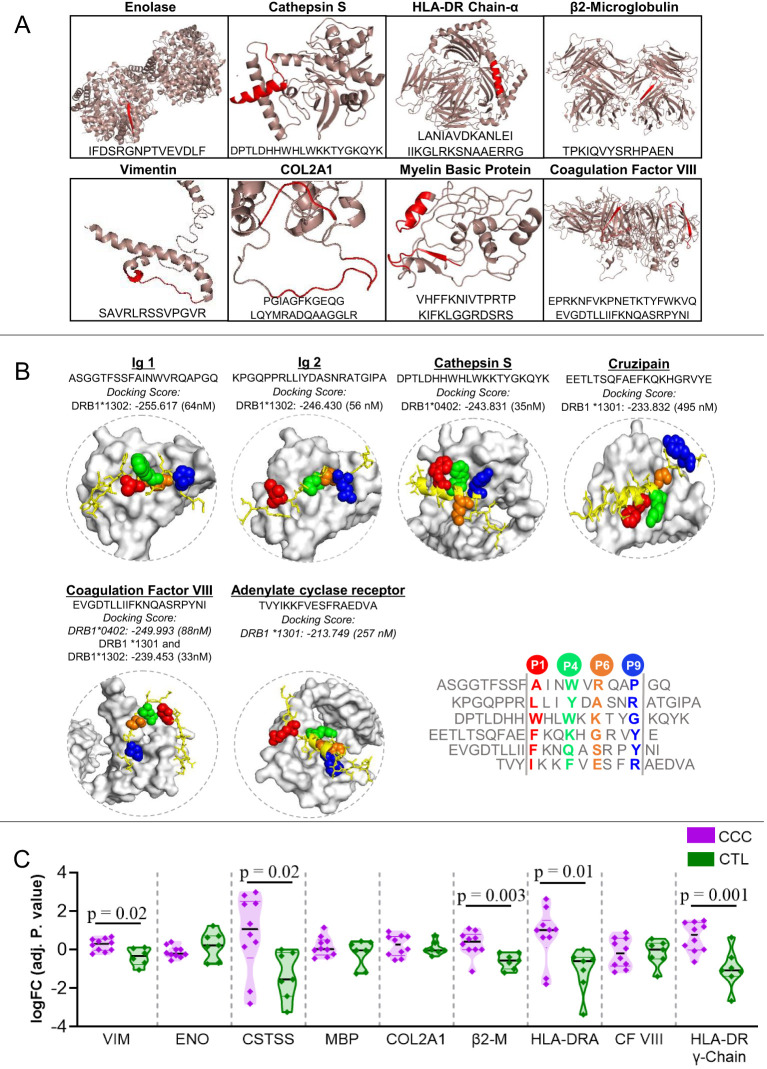
Three-dimensional visualization and interaction of antigens potentially recognized by the different classes of DERAA-bearing HLA-DRB1. **(A)** The 3D structures of human-proteins potentially recognized by HLA-DRB1 *0103, *0401, *1301 and *1302 alleles visualized using the PyMOL software (version 1.2r3pre). The location of the peptide recognized by HLA-DRB1 is highlighted in red. **(B)** Peptides bounds to the HLA-DRB1 interface visualized using as template structure PBD 1aqd to *13 allele and 1j8h to *04 allele. The HLA-DRB1 molecule is shown in white surface. The alignment of the peptide sequences in the core region is shown. The amino acids that interact with the protein pockets are colored in red (P1), green (P4), orange (P6) and blue (P9). The amino acids residues colored in yellow are the amino acids residues that bridge TCR contact. **(C)** Differently expressed genes in the Chagas heart tissue. Cardiac tissue transcripts from CCC patients (10 end-stage heart failure patients) and health subjects (7 heart tissues), available in Gene Expression Omnibus (accession number GEO 84796), were measured as described in Material and Methods. The expression of heart transcripts was expressed as logFC (adj. p value). Comparative analysis between CCC (chronic Chagas cardiomyopathy) and CTL (Control) was carried out by Student’s t-test according to data normality distribution. Significant differences with p < 0.05 are demonstrated. Ig1, Immunoglobulin 1; Ig2, Immunoglobulin 2; CTSS, Cathepsin S; MBP, Myelin Basic Protein; COL2A1, Collagen α-1; β2-M, β2-microglobulin; CF VIII, Coagulation factor VIII.

We conducted a comprehensive search of the IEDB database for *T. cruzi* and human peptides that are potentially recognized by the DERAA bearing HLA-DRB1 alleles: *0103, *0402, *1301 and *1302. Our analysis revealed a total of 532 peptides from 165 antigens within the human organism that have the potential to bind to the four HLA-DRB1 alleles. Specifically, 158 peptides from 81 antigens were predicted to bind to HLA-DRB1 *0103, 159 peptides from 35 antigens were predicted to bind to HLA-DRB1 *0402, 88 peptides from 25 antigens were predicted to bind to HLA-DRB1 *1301, and 127 peptides from 24 antigens were predicted to bind to HLA-DRB1 *1302. Promiscuous peptides, i.e., those that can be recognized by at least two of the HLA-DRB1 alleles were identified. **Table 2** shows the peptides that can putatively bind to specific HLA-DRB1 alleles. These peptides are derived from the following proteins: vimentin intermediate filament, enolase-alpha, cathepsin S, myelin basic protein (MBP), collagen 2 α-1, β2-microglobulin, HLA-DRA, and immunoglobulins. We also found invariant chain peptides (HLA-DR chain-γ or CD74) that can be recognized by these alleles, likely due to their natural binding to the peptide binding cleft, blocking the association of self-peptides or low-affinity peptides. **Figure 1A** exhibits representative images of the recognizable human proteins in their three-dimensional conformation, highlighting the location of peptides within the protein in red.

**Table 2:** Peptides derived from human antigens potentially recognized by the HLA-DRB1 *0103, *0402, *1301 and *1302 alleles.

Antigens	*0103 allele	*0402 allele	*1301 allele	*1302 allele
**Immunoglobulins**	KVQWKVDNALQSG	KPGQPPRLLIYDASNRATGIPA	SKNTVYLQIDSLRAEDTA	ASGGTFSSFAINWVRQAPGQ
	IQVSWLREGKQVGSG			
	KVQWKVDNALQSGN			
**Vimentin**	SAVRLRSSVPGVR SAVRLRSSVPGVR	SAVRLRSSVPGVR SAVRLRSSVPGVR		SAVRLRSSVPGVR SAVRLRSSVPGVR
**Enolase-alpha**	IFDSRGNPTVEVDLF IFDSRGNPTVEVDLF	IFDSRGNPTVEVDLF IFDSRGNPTVEVDLF	IFDSRGNPTVEVDLF IFDSRGNPTVEVDLF	
**Cathepsin S**			DPTLDHHWHLWKKTYGKQYK	DPTLDHHWHLWKKTYGKQYK DPTLDHHWHLWKKTYGKQYKE
**Myelin Basic Protein**			VDAQGTLSKIFKLGGRDSRS KIFKLGGRDSRSGS	NPVVHFFKNIVTPRTPPPSQ VHFFKNIVTPRTP
**Collagen 2 α-1**	PGIAGFKGEQGPKGE GKPGIAGFKGEQGPKG	PGIAGFKGEQGPKGE		
		LQYMRADQAAGGLR		
		LQYMRADQAAGGLR		
		QTGKPGIAGFKGEQGPKGEP		
		GKPGIAGFKGEQG		
		GKPGIAGFKGEQGPKG		
**β2-Microglobulin**			T PKIQVYSRHPAENGKSN	TPKIQVYSRHPAENG
			TPKIQVYSRHPAEN	TPKIQVYSRHPAENGKSN
			TPKIQVYSRHPAENG	
			TPKIQVYSRHPAENGK	
			TPKIQVYSRHPAENGKS	
**HLA-DR** **(α-Chain)**	LANIAVDKANLE	LANIAVDKANLEIMTKR IIKGLRKSNAAERRG		
	LANIAVDKANLEI			
	LANIAVDKANLEIM			
	LANIAVDKANLEIMT			
	NIAVDKANLEIM			
**HLA-II** **(γ-Chain)**		HHWLLFEMSRHSLE MHHWLLFEMSRHSLE WLLFEMSRHSLEQKP LPKPPKPVSKMRMATPLLQ	LPKPPKPVSKMRMATPLLMQ	TTAYFLYQQQGRLDK ATPLLMQALPMG LPKPPKPVSKMRM
**Coagulation Factor VIII**			EPRKNFVKPNETKTYFWKVQ EVGDTLLIIFKNQASRPYNI	EVGDTLLIIFKNQASRPYNI EPRKNFVKPNETKTYFWK

The names of the antigens are presented in bold.

Notably, we did not identify any *T. cruzi* peptides that were predicted to bind to any of these four HLA-DRB1 alleles. Thus, our findings suggest that HLA-DRB1 *0103, *0402, *1301 and *1302 alleles may be involved in the recognition of self-peptides in the context of *T. cruzi* infection.

### 3.2 Human peptides recognized by HLA-DRB1 alleles share similarity with *T. cruzi* peptides

Since no *T. cruzi* peptides recognized by the four classes of HLA-DRB1 were identified in the IEDB database, we investigated the similarity between the putative recognizable human peptides and parasite-derived peptides.

**Table 3** presents the identified peptides from *T. cruzi* with at least a 9-mer that present similarity with the self-peptides selected on the HLA-DRB1 alleles, including trans-sialidase (query cover 69%), CDC16 (query cover 61%), calpain (query cover 75%) and trypanothione (query cover 61%), among others. Furthermore, we found that a human α-enolase peptide exhibited a query cover of 86% with a parasite enolase peptide, while a cathepsin S peptide showed a query cover of 35% with a cruzipain-parasite peptide, and a COL2A1 peptide showed query cover of 30% with a trans sialidase-parasite peptide (**Table 3**).

**Table 3:** *Trypanosoma cruzi*-peptides similar to human peptides selected by DERAA-bearing MHC.

Human Peptide	HLA-DR alleles that recognizes	*T. cruzi* protein similarity	Query cover	Sequence alignment
**Immunoglobulins** ASGGTFSSFAINWVRQAPGQ	*1302	Calpain (RNF16885.1)	75%	SGGTFSS__FA I NW SGGTDSSSKFATNW
KVQWKVDNALQSG	*0103	Trans sialidase (EKG06737.1)	69%	WK -VDNALQS WKDLDNALQS
IQVSWLREGKQVGSG	*0103	Reverse transcriptase SLACS (PWV10075.1)	60%	VSWLREGKQ VSW_REGKQ
SKNTVYLQIDSLRAEDTA	*1301	Adenylate cyclase receptor (PWU87842.1)	83%	TVYLQ—IDSLRAEDTA TVYIKKFVESFRAEDVA
		CDC16 (EKG02051.1)	61%	VYLQIDSLRAE VY_ QIDPFRAE
		Glycosylphosphatidylinositol-specific phospholipase C (EKF26652.1)	61%	VYLQIDSLRAE VYLNI__LRAE
		Anion-transporting ATPase (KAF8281231.1)	94%	SKNTVYLQIDSL SK__YLAQIDSL
		Retrotransposon Hot spot protein (PWU93136.1)	50%	QIDSLR_AED QINSLRLAEE
KPGQPPRLLIYDASNRATGIPA	*0402	Rab1 small GTP-binding (RNC54059.1)	68%	RLL___IYDASNRATG__IP RLLQTAGIIEDASSRTTGGWIP
		Inositol-1,4,5-trisphosphate (IP3) 5-phosphatase (RNF22963.1)	45%	RLLIYDASNR RLLIHEASSR
		Trypanothione (EKG00422.1)	61%	GQPPRLLIYDASNR GHP_LLIYNVAS_R
**α-Enolase** IFDSRGNPTVEVDLF (NP_001419)	*1301 *0103 *0402	Enolase (EKG04398.1)	86%	IFDSRGNPTVEVDL ILDSRGNPTVEVEV
**Cathepsin S** DPTLDHHWHLWKKTYGKQYK (NP_004070.3)	*1301 *1302	Cruzipain (XP818579.1)	35%	DPTLDHHWHLWKKTYGKQYK EETLTSQFAEFKQKHGRVYE
**COL2A1** PGIAGFKGEQG (NP_001835.3)	*0103 *0402	Trans-Sialidase (EKG06737.1)	30%	PGIAGFKGEQGP PAGASEEGSRGD

Promiscuous human peptides similar to *Trypanosoma cruzi-*peptides. BLAST of human-peptides recognized by at least two of the HLA-DRB1 alleles were submitted to BLAST in order to identify similarity to *Trypanosoma cruzi* (taxid:5693). Query cover indicates percentage of sequence submitted to the BLAST that is covered by the alignment. Accession number to Genebank of each protein was indicated. Letters highlighted in red indicate amino acids that are different from the human peptide.

These findings indicate that DERAA-bearing HLA-DRB1 alleles putatively recognize self-peptides with similarity to *T. cruzi* peptides, suggesting that the parasite may induce a cross-reactive response to these human proteins, which may play a crucial role in CCC pathogenesis.

Analysis to predict the binding affinity of human and *T. cruzi* peptides to HLA-DRB1 *0103, *0402, *1301 and *1302 alleles, as shown in **Supplementary Table 2**. Our findings indicate that the immunoglobulin 1 (ASGGTFSSFAINWVRQAPGQ) and coagulation factor VIII (EVGDTLLIIFKNQASRPYNI) human peptides have binding affinity to all HLA-DRB1 classes, although varying in affinity. The immunoglobulin 1 (ASGGTFSSFAINWVRQAPGQ) peptide displayed intermediate binding affinity to *0103, *0402 and *1301 alleles and high binding affinity to *1302 allele, while the coagulation factor (EVGDTLLIIFKNQASRPYNI) peptide displayed intermediate binding affinity to *0103 allele and high binding affinity to *0402, *1301 and *1302 alleles. The HLA-DRB1 *0402 allele showed intermediate binding affinity to HLA-DR α-chain (IIKGLRKSNAAERRG) and immunoglobulin 2 (KPGQPPRLLIYDASNRATGIPA) peptides, and strong binding affinity to cathepsin S peptide. The HLA-DRB1 *1301 allele displayed intermediate binding affinity to β2-microglobulin, cathepsin S, immunoglobulins, (IQVSWLREGKQVGSG and KPGQPPRLLIYDASNRATGIPA), and coagulation factor VIII (EPRKNFVKPNETKTYFWKVQ) peptides. The HLA-DR α-Chain peptide (IIKGLRKSNAAERRG) displayed strong binding affinity to *1301 allele. The HLA-DRB1 *1302 allele presented intermediate binding affinity to myelin basic protein (MBP) (VHFFKNIVTPRTP), HLA-DR α-Chain (LANIAVDKANLEI and IIKGLRKSNAAERRG), immunoglobulin (KVQWKVDNALQSG), and coagulation factor VIII (EPRKNFVKPNETKTYFWKVQ) peptides. Lastly, immunoglobulin 2 peptide (KPGQPPRLLIYDASNRATGIPA) had high binding affinity to *1302 allele (**Supplementary Table 2**). Collectively, our results suggest that the HLA-DRB1 *13 allele is closely associated with self-peptide recognition.

Regarding the *T. cruzi*-peptides, only HLA-DRB1*13 selectively binds to parasite-derived peptides that are similar to human peptides. Specifically, HLA-DRB1 *1301 allele displays intermediate binding affinity to adenylate cyclase receptor and cruzipain, while HLA-DRB1 *1302 allele shows intermediate binding affinity only to adenylate cyclase receptor. The remaining *T. cruzi*-peptides exhibit low binding affinity to all HLA-DRB1 alleles tested (**Supplementary Table 2**). These data suggest that HLA-DRB1 *13 may play a crucial role in recognizing self-peptides, as well as potential cross-reactive *T. cruzi*-peptides that resemble human peptides.

To confirm the interaction potential between self and non-self peptides and HLA alleles, we used molecular docking for those peptides with strong (<100 nM) or intermediate (between 100 to 1000 nM) binding affinity to HLA-DRB1. Molecular docking predicts the binding mode and affinity between peptide and HLA-DRB1 (48). **Supplementary Table 3** shows that cathepsin S, immunoglobulins 1 and 2 and coagulation factor VIII have the highest docking scores. The parasite peptides, adenylate cyclase receptor and cruzipain, which had intermediate binding affinity to HLA-DRB1 *13 allele, showed a strong docking score (**Supplementary Table 3**).

**Figure 1B** displays the 3D structure of peptides that strongly or moderately bind to HLA-DRB1 alleles, namely immunoglobulins 1 and 2 (ASGGTFSSFAINWVRQAPGQ and KPGQPPRLLIYDASNRATGIPA, respectively), cathepsin S, cruzipain, coagulation factor VIII, and adenylate cyclase receptor. These peptides were selected based on their high docking scores (<1000 nM) obtained through molecular docking analysis. The 3D visualization reveals that they bind to the peptide binding cleft, indicating that these HLA-DRB1 alleles may preferentially bind to these peptides, suggesting a mechanism of dominant peptide selection (**Figure 1B**).

### 3.3 Predicted human and *T. cruzi-*peptides have immunogenic properties capable of triggering modulatory and inflammatory cytokine secretion using *in silico* models

We predicted the immunogenicity and functional response of the different peptides using the CD4+ T Cell Immunogenicity prediction tool (http://tools.iedb.org/CD4episcore) from the IEDB. **Table 4** shows that human peptides with the highest immunogenicity score were MBP (57.5 score), HLA-DR α-Chain (29.8 score) and vimentin (43.4 score), indicating their potential to induce production of IL-4 and IFN-γ by T cells, respectively. Immunoglobulin 1 (52.6 score) and coagulation factor VIII (72.1 score), which bind to all HLA-DRB1 classes, were classified as immunogenic and displayed the potential to induce both IFN-γ and IL-4 by T cells. **Table 4** also lists other immunogenic self-peptides with intermediate binding affinity, such as β2-microglobulin, immunoglobulins and coagulation factor VIII, which displayed immunoregulatory potential.

**Table 4:** Immunogenicity and potential of cytokine induction of human-derived and *Trypanosoma cruzi-*derived peptides.

Antigen	Peptide	Immun.	IFN-γ	IL4
Vimentin	SAVRLRSSVPGVR	**43.4**	**0.289**	**-**0.27
Enolase-α	IFDSRGNPTVEVDLF	81.8	-0.556	-0.19
MBP	VHFFKNIVTPRTP	**25.1**	-0.520	**1.0**
	KIFKLGGRDSRS	**57.5**	**0.207**	**0.55**
β2M	TPKIQVYSRHPAEN	**64.9**	-0.447	**0.22**
HLA-DR α-Chain	LANIAVDKANLEI	**48.9**	**0.295**	**0.41**
	IIKGLRKSNAAERRG	**29.8**	**0.437**	-0.06
HLA-II γ-Chain	ATPLLMQALPMG	**50.3**	-0.123	**0.26**
	HHWLLFEMSRHSLE	**48.1**	-0.400	**0.27**
	LPKPPKPVSKMRM	89.4	-1.012	**0.25**
Cathepsin S	DPTLDHHWHLWKKTYGKQYK	81.3	**1.216**	**0.25**
COL2A1	PGIAGFKGEQG	90.6	**0.903**	**0.63**
	LQYMRADQAAGGLR	**46.4**	**0.014**	**0.21**
Immunoglobulins	KVQWKVDNALQSG	–	-0.385	**0.29**
	IQVSWLREGKQVGSG	65.9	-0.032	**0.24**
	ASGGTFSSFAINWVRQAPGQ	**52.6**	**0.999**	**0.93**
	KPGQPPRLLIYDASNRATGIPA	90.2	**1.677**	-0.70
	SKNTVYLQIDSLRAEDTA	**46.1**	**0.247**	-0.30
Coagulation Factor VIII	EPRKNFVKPNETKTYFWKVQ	**72.1**	**0.290**	**0.38**
	EVGDTLLIIFKNQASRPYNI	**46.5**	**0.745**	-0.13
Calpain	SGVTFGSALSQLTGGITFAIDW	**65.6**	**1.346**	0.06
Trans-sialidase	WKDLDNALQS	**72.2**	-0.691	**0.28**
ACR	TVYIKKFVESFRAEDVA	**50.72**	-0.306	**0.30**
CDC16	VYQIDPFRAE	**47.8**	**0.372**	**0.37**
Rab1 small GTP-binding protein	RLLQTAGIIEDASSRTTGGWIP	80.5	-0.857	-0.44
Enolase	ILDSRGNPTVEVEV	79.2	-0.115	-0.15
Cruzipain	EETLTSQFAEFKQKHGRVYE	76.7	**0.492**	**0.37**
GPI-PLC	VYLNILRAE	**32.3**	**0.761**	0.15
Anion-transporting ATPase	SKYLAQIDSL	**57.1**	-0.966	**0.28**
Retrotransposon Hot spot protein	QINSLRLAEE	**65.8**	-0.847	-0.11
Ins(1,4,5)P3	RLLIHEASSR	**35.6**	-0.238	**0.28**
Trypanothione	GHPLLIYNVASR	**47.5**	-0.379	-0.17
Reverse Transcriptase	VSWREGKQRVLTEEL	81.1	**0.111**	0.17

Score < 73: Immunogenic peptide. IFN-γ: Threshold ≥ 0.0 are peptides potentially IFN-γ inducers. IL4: Threshold ≥ 0.2 are peptides potentially IL4 inducers. Immun., Immunogenicity; MBP, Myelin Basic Protein; β2M, β2-Microglobulin; ACR, Adenylate cyclase receptor; GPI-PLC, Glycosylphosphatidylinositol-specific phospholipase C; Ins(1,4,5)P3, Inositol-1,4,5-trisphosphate (IP3) 5-phosphatase. Bold indicates that peptides in on score/threshold to immunogenicity or potential to induce IFN or IL4 cytokines.

Among *T. cruzi*-peptides, adenylate cyclase receptor, anion-transporting ATPase and Ins (1,4,5)P3 peptides were classified as immunogenic (<73 score) and potential to induce IL-4 production (≥ 0.2 score) by T lymphocytes (**Table 4**). These data suggest that HLA-DRB1 classes may select dominant *T. cruzi*-peptides and self-peptides with potential to induce cytokine production which will be crucial for orchestrating the immune response.

### 3.4 Human proteins containing peptides recognized by the HLA-DRB1 alleles are differentially expressed in cardiac tissue from CCC patients

Upon identifying specific self-peptides that can be recognized by HLA-DRB1 alleles, we evaluated whether the genes that code for these proteins are differentially expressed in cardiac tissue from CCC patients, as compared to non-Chagas individuals. The data show increased expression of vimentin (p = 0.02), cathepsin S (p =0.02), β2-microglobulin (p =0.003), HLA-DRA (p =0.01), and HLA-DR γ-Chain (p =0.001) transcripts in CCC cardiac tissue compared to that of healthy donors. However, enolase, MBP, COL2A1 and coagulation factor VIII genes did not show differential expression. Immunoglobulin genes were not found in the cardiac transcripts data (**Figure 1C**).

### 3.5 CD4+ T-cells from CCC patients, but not from IND patients, respond to both human and *T. cruzi-*peptides

After conducting *in silico* analysis of human and parasite peptides potentially recognized by DERAA-bearing DRB1 alleles, we evaluated the potential of these peptides to induce activation and proliferation of human lymphocytes *in vitro*. To determine this, we measured the expression of CD69 in two subpopulations of T lymphocytes labeled with CFSE: one that did not proliferate (R1) and the other that proliferated (R2). Previous studies have shown that *in vitro* T-cell proliferation requires continuous 10-24h TCR signaling, which leads to a lymphocyte population with a typical blast appearance due to increased size (49, 50). Thus, we performed this analysis on total lymphocytes (small + blast lymphocytes gated together), small lymphocytes (small lymphocytes gated), and blasts (large lymphocytes gated) (**Supplementary Figure 1A**). Additionally, we ensured that the cells in the gate of total, small and blast T-cell populations did not express CD14+, indicating that CD14+ monocyte populations were not considered (**Supplementary Figure 1B**).

**Figure 2:**
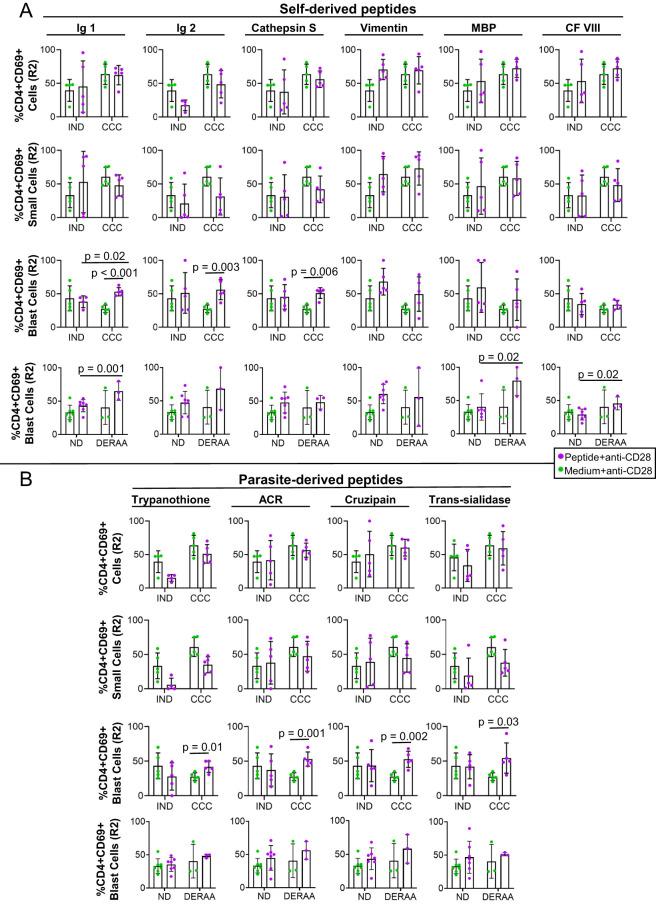
Frequency of CD4+CD69+ total lymphocytes, small and blast cells after stimulation with self-derived and *T. cruzi*-derived peptides. **(A)** Frequency (%) of CD4+CD69+ T lymphocytes, small and blast cells proliferating (R2) from indeterminate (IND, n=5) and chronic Chagas cardiomyopathy (CCC, n=5) patients stimulated with medium and anti-CD28 or self-peptides and anti-CD28. **(B)** Frequency (%) of CD4+CD69+ T lymphocytes, small and blast cells proliferating (R2) from indeterminate (IND, n=5) and chronic Chagas cardiomyopathy (CCC, n=5) patients stimulated with medium and anti-CD28 or parasite-derived peptides and anti-CD28. The analysis was performed as described in the materials and methods for comparison between the groups. Significant differences with p < 0.05 are demonstrated.

No self-derived, nor parasite-derived, peptides led to significant changes in the frequency of CD4+CD69+ cells within the total lymphocyte population nor small cells (**Figures 2A, B**, top two rows). However, analysis within the blast cell population showed that Immunoglobulin 1 and 2 and cathepsin S-derived peptides induced a higher frequency of CD4+CD69+ in CCC but not in IND (**Figure 2A**, third row). All parasite-derived peptides also induced a higher frequency of CD4+CD69+ blasts in CCC but not in IND (**Figure 2B**, third row).

We conducted HLA typing on all donors and observed that the *1301 allele of HLA-DRB1 was expressed by 40% of CCC donors and 20% of IND donors (**Table 1**). When comparing the DERAA-bearing patients with the DERAA negative patients, we observed a higher frequency of CD4+CD69+ blast cells in response to stimulation with peptides derived from immunoglobulin 1, MBP, and coagulation factor VIII in the former, as depicted in **Figure 2A** (bottom panels). Notably, the frequency of CD4+CD69+ blast cells remained unaltered when exposed to parasite-derived peptides, both in non-DERAA and DERAA donors (**Figure 2B**, bottom panels).

We also evaluated the potential to induce activation and proliferation of CD8+CD69+ and CD19+HLA-DR+ lymphocytes after stimulation with self and non-self peptides. Our analysis showed that peptides did not change the frequency of CD8+CD69+ and CD19+HLA-DR+ total lymphocytes, small and blast cells (**Supplementary Figures 2**, **3**, respectively).

**Figure 3:**
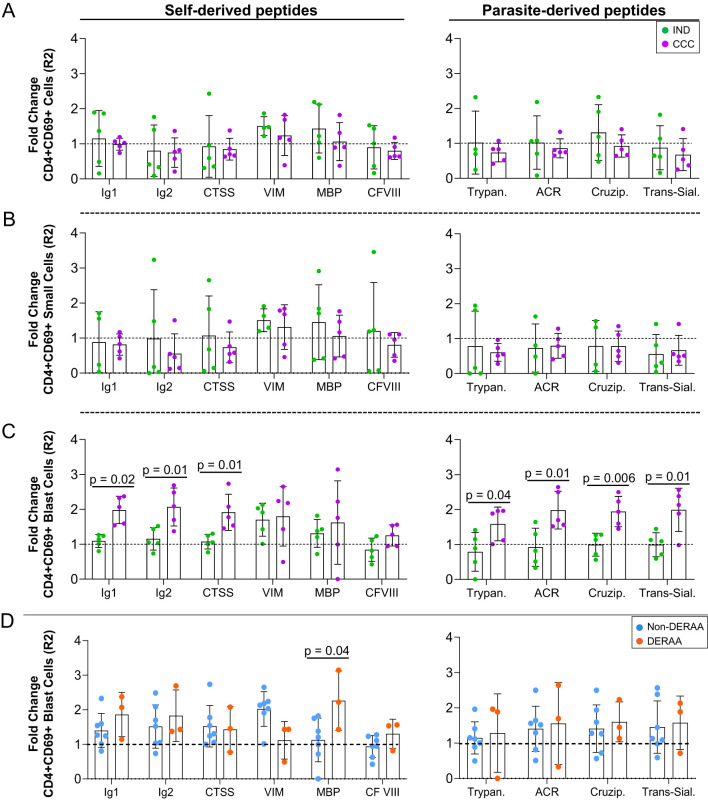
Proliferation index of CD4+CD69+ cells stimulated with self and non-self-peptides. **(A)** Proliferating index of CD4+CD69+ T lymphocytes, **(B)** small and **(C)** blast cells from indeterminate (IND, n=5) and chronic Chagas cardiomyopathy (CCC, n=5) patients stimulated with self-derived and parasite-derived peptides plus anti-CD28. The analysis was performed as described in the materials and methods for comparison between the groups. **(D)** Proliferating index of CD4+CD69+ blast cells indicating DERAA-HLA-DRB1 bearing patients as orange dots and non-DERAA as blue dots. The statistical analysis was performed as described in the material and methods for comparisons between the groups. Significant differences with p < 0.05 are demonstrated. Ig1, Immunoglobulin 1; Ig2, Immunoglobulin 2; CTSS, Cathepsin S; VIM, Vimentin; MBP, Myelin basic protein; CF VIII, Coagulation factor VIII; Trypan, Trypanothione; ACR, Adenylate cyclase receptor; Cruzip., Cruzipain; Trans-Sial., Trans-Sialidase.

The proliferation index of CD4+CD69+ cells (R2) was determined by analysis of the stimulated/non-stimulated cultures and comparing between IND and CCC. While self and non-self peptides had no statistically significant effect on the proliferation index of CD4+CD69+ total and small cells (**Figures 3A, B**), the proliferation index of CD4+CD69+ blast cells was higher in CCC than in IND in the presence of immunoglobulin 1, immunoglobulin 2, cathepsin S and all parasite-derived peptides (**Figure 3C**). This finding reaffirms that CD4+ T cells from CCC but not IND respond to the self and parasite-derived peptides. When the proliferation index of CD4+CD69+ cells was compared between non-DERAA and DERAA donors, we observed that despite a slight increase in the response of DERAA compared to non-DERAA patients (except for vimentin), only the MBP peptide significantly increased the proliferation index of CD4+CD69+ blast cells from DERAA (**Figure 3D**). This MBP peptide is highly conserved in MBP from different animal species (**Supplementary Table 4**).

### 3.6 Soluble mediators involved in cardiac fibrotic remodeling are associated with the proliferation of CD4+CD69+ blast cells stimulated with self and non-self peptides

**Figure 4:**
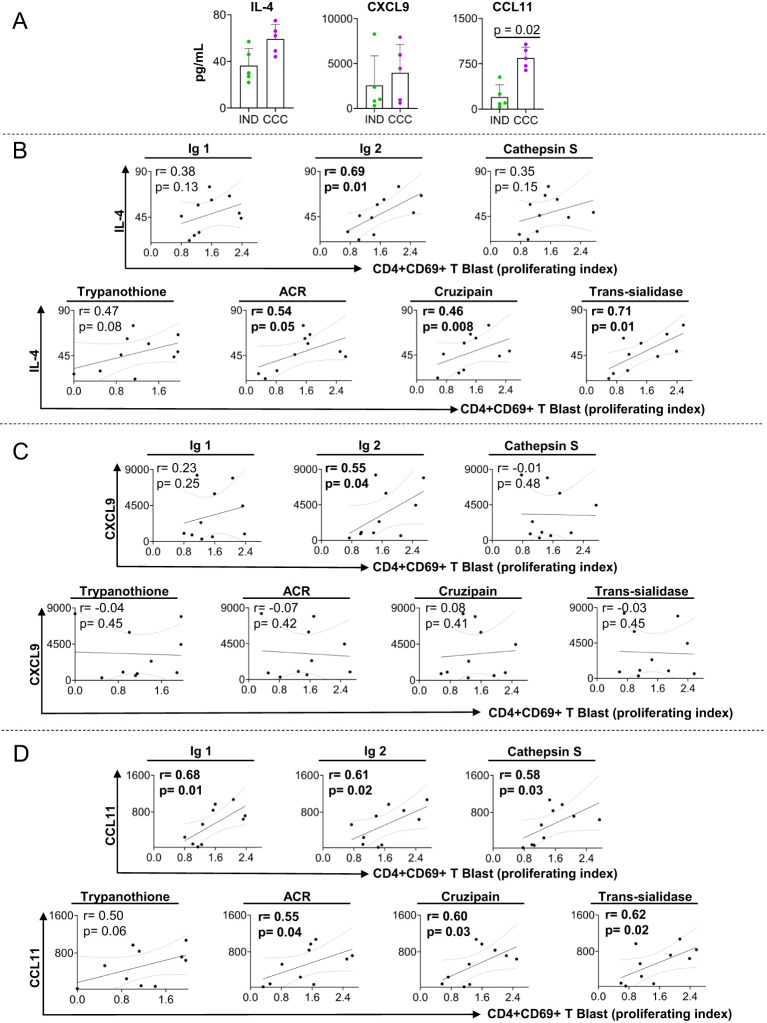
Plasma level of soluble mediators and correlation analysis between proliferation index of CD4+CD69+ blast cells and cardiac function markers. **(A)** levels of IL-4, CXCL9 and CCL11 in plasma from indeterminate (IND) and chronic Chagas cardiomyopathy (CCC) patients. **(B)** Correlation between the proliferation index of CD4+CD69+ blast cells from indeterminate and chronic Chagas cardiomyopathy patients stimulated with self-derived peptides (immunoglobulins 1 and 2 and cathepsin S), parasite-derived peptides (Trypanothione, Adenylate cyclase receptor (ACR), Cruzipain and Trans-Sialidase) and IL-4. **(C)** Correlation between the proliferation index of CD4+CD69+ blast cells from indeterminate and chronic Chagas cardiomyopathy patients stimulated with self-derived peptides (immunoglobulins 1 and 2 and cathepsin S), parasite-derived peptides (Trypanothione, ACR (Adenylate cyclase receptor), Cruzipain and Trans-Sialidase) and CXCL9. **(D)** Correlation between the proliferation index of CD4+CD69+ blast cells from indeterminate and chronic Chagas cardiomyopathy patients stimulated with self-derived peptides (immunoglobulins 1 and 2 and cathepsin S), parasite-derived peptides (Trypanothione, ACR (Adenylate cyclase receptor), Cruzipain and Trans-Sialidase) and CCL11. Parametric data were analyzed using Pearson’s correlation test and non-parametric data were analyzed using Spearman’s test. Significant differences with p < 0.05 are demonstrated.

To further characterize the immune profile of the patients and the association with proliferation induced by DERAA-selected peptides, we measured the levels of chemokines, pro-and anti-inflammatory cytokines, as well as growth factors in the plasma of the same IND and CCC subjects. We observed a clear trend of IL-4 and CXCL-9 levels to be higher in CCC as compared to IND, and a significant increase in CCL11 levels in plasma from CCC than IND (**Figure 4A**). Other factors measured did not show any statistical significance between groups (**Supplementary Figure 4**).

We investigated the correlation between the altered soluble mediators and the proliferation index of CD4+CD69+ blast cells induced by peptides that led to distinct response in CCC and IND to better understand their systemic relationship. We observed that IL-4 was associated with proliferating index of CD4+CD69+ blast cells stimulated with immunoglobulin 2, adenylate cyclase receptor (ACR), cruzipain and trans-sialidase (**Figure 4B**). CXCL9 was positively associated with the proliferating index of CD4+CD69+ blast cells stimulated with immunoglobulin 2 (**Figure 4C**), but no correlation was observed with parasite-derived antigens (**Figure 4C**). Furthermore, we found a positive association between CCL11 and the proliferating index of CD4+CD69+ blast cells stimulated with immunoglobulins 1 and 2, cathepsin S adenylate cyclase receptor, cruzipain and trans-sialidase (**Figure 4D**).

### 3.7 Proliferation of CD4+CD69+ blast cells correlate with heart function and prognosis in Chagas disease patients

**Figure 5:**
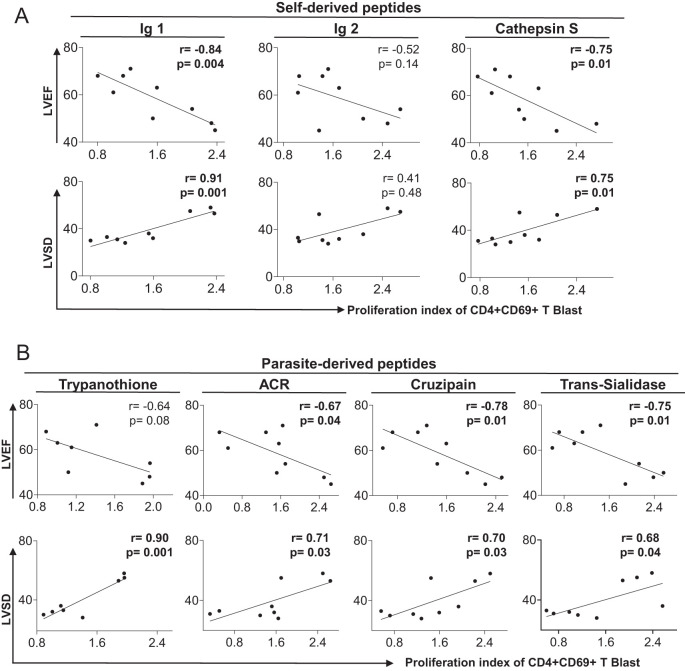
Correlation between clinical parameters of cardiac dysfunction and proliferative response to self- and parasite-derived DERAA-selected peptides. **(A)** Correlation between the proliferation index of CD4+CD69+ blast cells from indeterminate and chronic Chagas cardiomyopathy patients stimulated with self-derived peptides (immunoglobulins 1 and 2 and cathepsin S) and left ventricular ejection fraction (LVEF) or left systolic diameter (LVDD). **(B)** Correlation between the proliferation index of CD4+CD69+ blast cells from indeterminate and chronic Chagas cardiomyopathy patients stimulated with parasite-derived peptides (Trypanothione, Adenylate cyclase receptor (ACR), Cruzipain and Trans-Sialidase) and left ventricular ejection fraction (LVEF) or left ventricular systolic diameter (LVDD). Parametric data were analyzed using Pearson’s correlation test and non-parametric data were analyzed using Spearman’s test. Significant differences with p < 0.05 are demonstrated.

To determine the relationship between heart function and the proliferation index of CD4+CD69+ blast cells stimulated with self and non-self peptides, we performed correlation analysis between the proliferation index to the stimulating peptides and the left ventricular ejection fraction (LVEF – higher values indicate better ventricular contractility and systolic function) and the ventricular end-systolic diameter (LVSD – higher values indicate increased dilation with impaired function), two echocardiographic parameters of cardiac function (51). Our findings show an inverse correlation between LVEF and the proliferation index of CD4+CD69+ blast cells stimulated with immunoglobulin 1, cathepsin S (**Figure 5A**), ACR, cruzipain and trans-sialidase (**Figure 5B**). Moreover, we observed a positive association between LVSD and immunoglobulin 1, cathepsin S (**Figure 5A**), trypanothione, ACR, cruzipain and trans-sialidase (**Figure 5B**). These data suggest that the high proliferation of CD4+CD69+ blast cells induced by self and non-self peptides is associated with worse heart function and severity of Chagas disease.

### 3.8 scRNAseq analysis revealed that activated memory CD4+ T-cells derived from Chagas patients displaying the DERAA motif exhibit a cytotoxic, inflammatory and proapoptotic profile

**Figure 6:**
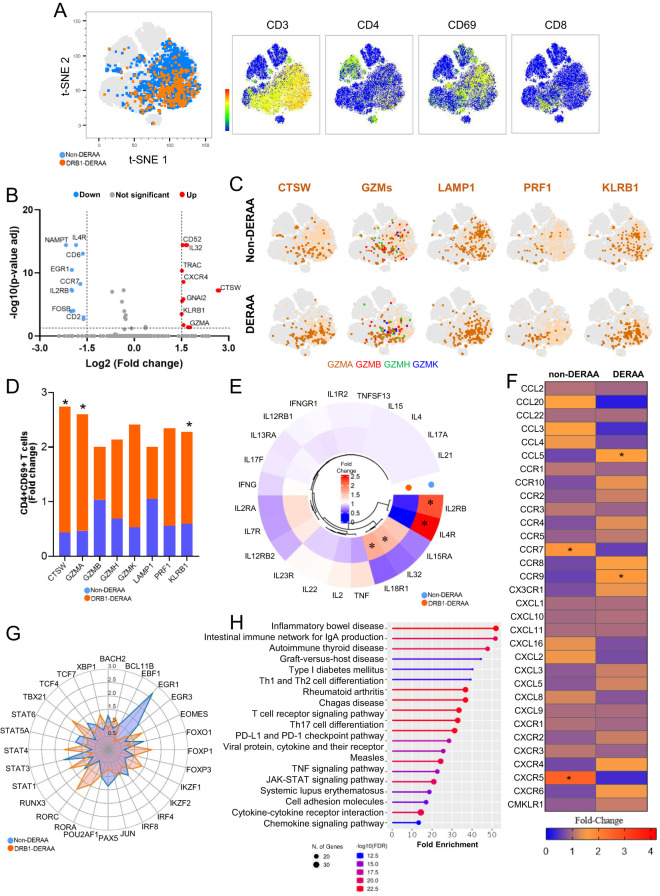
Differentially expressed genes in activated CD4+ T-cells from individuals with indeterminate and chronic Chagas cardiomyopathy. **(A)** Density of CD3, CD4, CD69 and CD8 gene expression in populations of CD4+CD3+CD69+ T cells formed by unsupervised analysis employing the tSNE algorithm. CD4+CD3+CD69+ T cells from non-DERAA donors are indicated in blue, while cells from donors expressing DRB1-DERAA HLA (*0103, *0402, *1301 and *1302 alleles) are indicated in orange. **(B)** Volcano Plot of patients with chronic Chagas cardiomyopathy compared to those with indeterminate form. Upregulated genes (Log2 Fold-change > 1.5 and Bonferroni-adjusted *p <*0.05) are shown in red, and downregulated genes (Log2 Fold-change < -1.5 and Bonferroni-adjusted *p <*0.05) are shown in blue. Genes without significant differential expression (Log2 Fold-Change between 1.5 and -1.5 and Bonferroni-adjusted *p >*0.05) are shown in grey. **(C)** Distributions of CTSW, GZMs (Granzyme A, B, H and K), LAMP1, PRF1 and KLRB1 gene expression in CD4+CD69+ T cells from non-DERAA and DERAA donors. **(D)** Bar graph demonstrating the expression of genes involved in cytotoxic processes in activated CD4+ T cells from non-DERAA (blue) and DRB1-DERAA (orange) donors. **(E)** Heatmap demonstrating the gene expression of cytokines and cytokine receptors, and **(F)** chemokines and chemokine receptors in activated CD4+ T cells from non-DERAA and DRB1-DERAA donors. **(G)** Radar graph of transcription factor gene expression in activated CD4+ T cells from non-DERAA (blue) and DRB1-DERAA (orange) donors. **(H)** Signaling pathways enriched in activated CD4+ T cells from donors expressing the HLA-DRB1 alleles. This analysis was performed considering only upregulated and downregulated DEGs. *Indicate differentially expressed genes (DEGs) upregulated (Log2 Fold-change > 1.5) and downregulated DEGs (Log2 Fold-change < -1.5) and Bonferroni-adjusted p value <0.05 are considered significant.

To gain insight into the diverse spectrum of CD4+CD69+ T-cells in donors expressing or not the alleles that contain the DERAA motif (DERAA or non-DERAA, respectively), we conducted scRNA-seq analyses on 29,094 CD4+ T cells from Chagas patients. Unsupervised clustering of DERAA and non-DERAA patients revealed a population of CD4+CD3+ T cells co-expressing CD69 (**Figure 6A**). We found 24 DEGs in CD3+CD4+CD69+ cells from DERAA compared to non-DERAA, of which 13 were up-regulated and 11 down-regulated (**Figure 6B**). Strikingly, CD3+CD4+CD69+ cells from DERAA donors exhibited a significant upregulation of cytotoxicity-associated genes, such as CTSW, GZMA and KLRB1 (**Figures 6C, D**). In addition, cells from DERAA patients displayed higher expression of IL32 and IL18R1 (**Figure 6E**), and heightened expression of chemotactic genes CCL5 and CCR9, whereas non-DERAA cells exhibited elevated expression IL2RB and IL4R genes and chemotactic genes CCR7 and CXCR5 (**Figures 6E, F**). Consistent with the observed genotypic differences, CD3+CD4+CD69+ cells from DERAA donors demonstrated pronounced expression of EOMES, STAT1, and STAT4 genes, while CD3+CD4+CD69+ cells from non-DERAA exhibited elevated expression of IRF4, EGR1 and STAT3 (**Figure 6G**), indicating an inflammatory and cytotoxic profiles in cells from DERAA patients compared to non-DERAA. When we evaluated all DEGs in cells from DERAA donors an enrichment of signaling pathways closely tied to activation and differentiation of immune cells, apoptosis, as well as enrichment of chemotaxis, cytokine-mediated and TCR signaling process were observed (**Figure 6H**).

**Figure 7:**
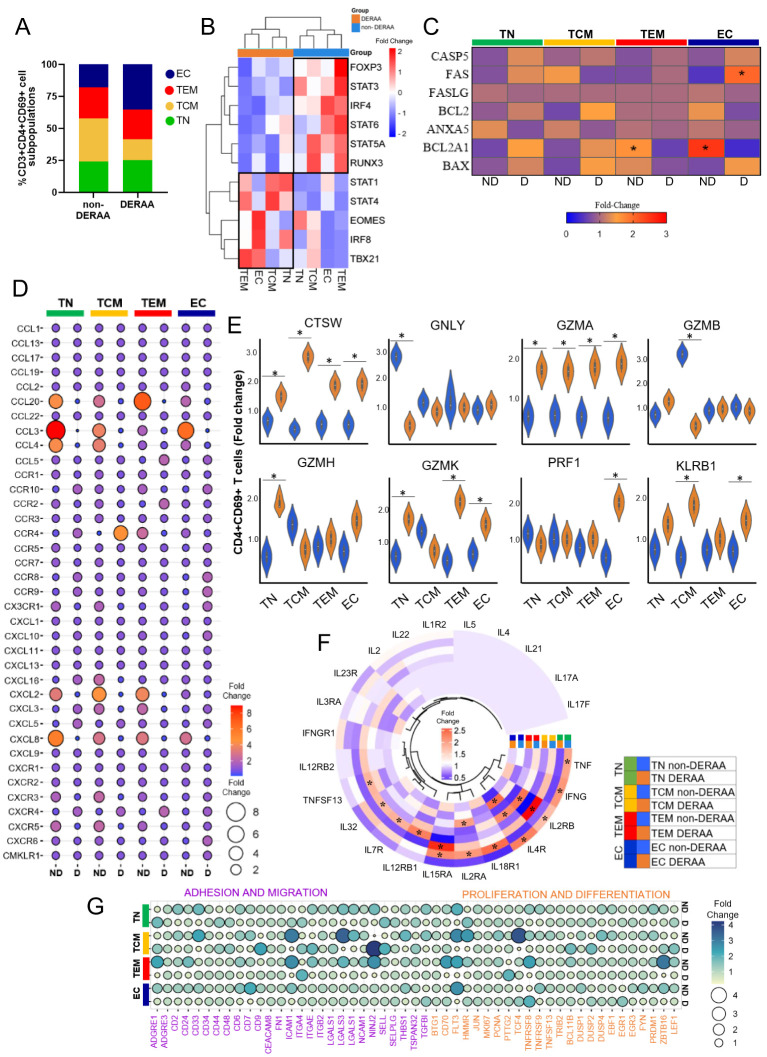
Differentially expressed genes in memory CD4+ T cells from patients with Chagas asymptomatic and chronic Chagas cardiomyopathy. **(A)** Graph showing the frequency of T naïve (TN, CCR7+CD45RA+), T central memory (TCM, CCR7+CD45RA-), T effector memory (TEM, CCR7-CD45RA-) and T effector (CCR7-CD45RA+) among CD4+CD69+ T cells from non-DERAA and DERAA donors. **(B)** Heatmap displaying transcription factor gene expression from non-DERAA (blue, n=8) and DERAA (orange, n=9) donors. **(C)** genes involved in apoptosis pathway in CD4+CD69+ T cell subpopulations from non-DERAA (ND) and DERAA **(D)** donors. **(D)** Dot plot of the mean expression of chemokines and chemokine receptors, in CD4+CD69+ T cell subpopulations from non-DERAA (ND) and DERAA **(D)** donors. **(E)** Violin plots show the expression of cathepsin W (CTSW), granulysin (GNLY), Granzyme (GZM) A, GZMB, GZMH, GZMK, perforin (PRF1) and KLRB1 in memory CD4+CD69+ T cells from non-DERAA (blue) and DERAA (orange) donors. **(F)** Heatmap of the mean expression of cytokines and cytokine receptors in CD4+CD69+ T cell subpopulations from non-DERAA (orange) and DERAA (blue) donors. **(G)** Heatmap of the mean expression of adhesion and migration (purple), proliferation and differentiation (orange) markers in CD4+CD69+ T cell subpopulations from non-DERAA (ND) and DERAA **(D)** donors. *Upregulated genes (Log2 Fold-change > 1.5) and downregulated genes (Log2 Fold-change < -1.5) and Bonferroni-adjusted p value <0.05 are considered significant.

Further characterizing the CD3+CD4+CD69+ T cells based on differentiation states, we categorized the CD4+CD69+ T cells from DERAA and non-DERAA patients into T naïve (CD45RA+CCR7+, TN), central memory (CD45RA-CCR7+, TCM), effector memory (CD45RA-CCR7-, TEM), and effector cells (CD45RA+CCR7-, EC) (**Figure 7A**). The heatmap analysis comparing the four subpopulations of CD4+CD69+ T cells revealed that transcription factor genes characteristics of regulatory T cells, such as FOXP3, STAT3, IRF4, STAT6, STA5A, and RUNX3 were strongly expressed in cells from non-DERAA patients, while cells from DERAA exhibited strong expression of EOMES, TBX21, STAT1, STAT4, and IRF8, which are present in T cells with inflammatory and cytotoxic activity (**Figure 7B**). Also, TEM and EC cells from non-DERAA up regulated expression of BCL2A1 anti-apoptotic gene, while only EC cells from DERAA-donors upregulated the expression of FAS genes, which are involved in the proapoptotic process (**Figure 7C**).

**Figure 8:**
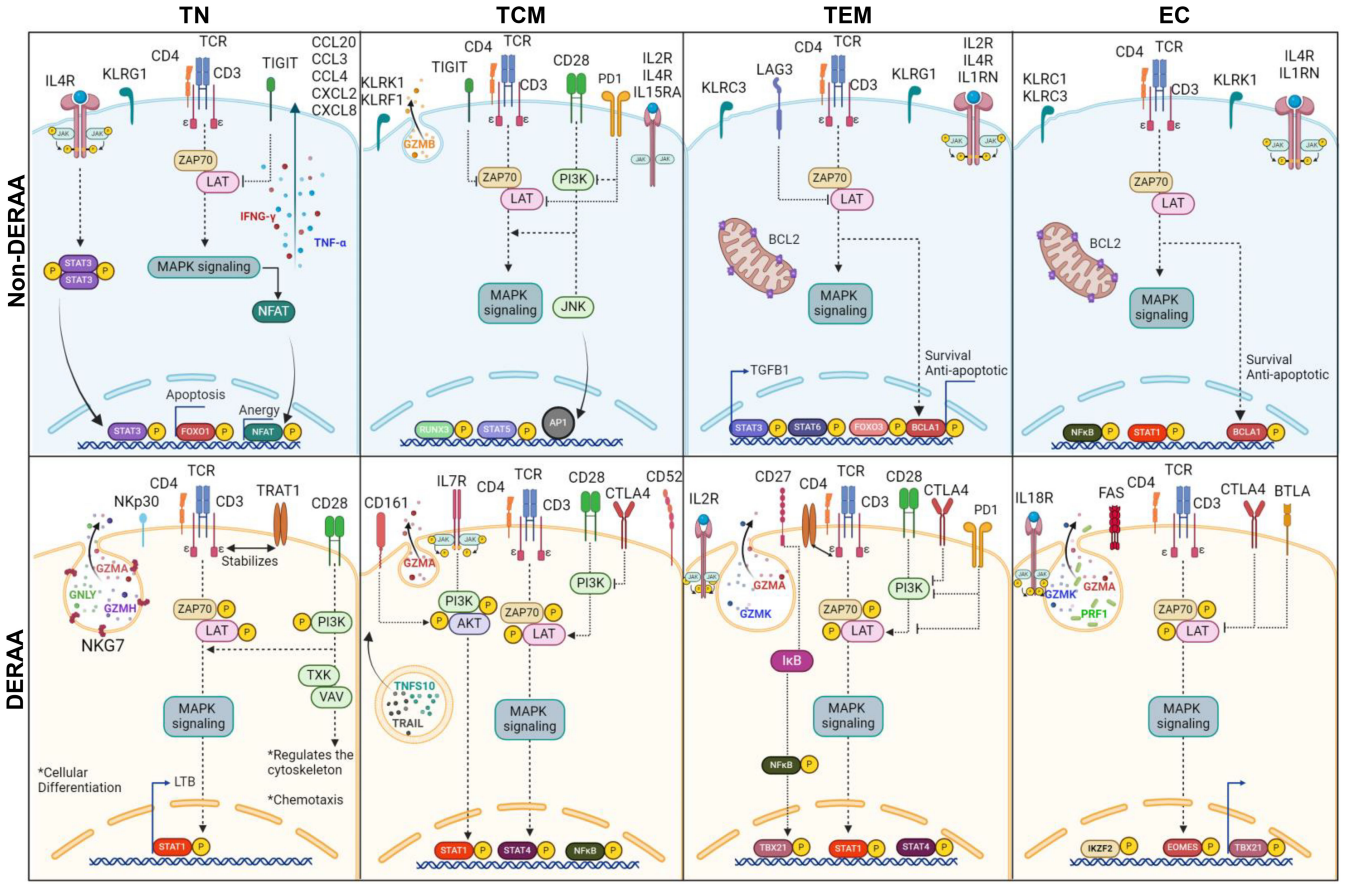
Schematic representation of differentially expressed genes in memory CD4+ T cells from donors expressing HLA-DRB1 alleles. Illustration of dichotomous gene expression pattern in distinct CD4+CD69+ T cell subsets, including T naïve (TN, CCR7+CD45RA+), T central memory (TCM, CCR7+CD45RA-), T effector memory (TEM, CCR7-CD45RA-) and T effector (CCR7-CD45RA+) cells from Non-DERAA (Blue panel) and DERAA donors (Orange panel). TN CD4+CD69+ from non-DERAA donors exhibit upregulation of TNF-α and IFN-γ cytokines. Additionally, there is an upregulation of IL4R, STAT3, genes inhibiting T cell receptor (TCR) signaling pathway (TIGIT), suggesting potential regulatory roles, and chemotactic genes (CCL20, CCL3, CCL4, CXCL2 and CXCL8). In contrast, TN CD4+CD69+ cells from DERAA donors demonstrate upregulation of genes contributing to the TCR signaling pathway (TRAT1, PI3K), costimulatory molecule (CD28), and genes crucial for cellular differentiation (LTB, STAT1). TCM CD4+CD69+ cells from non-DERAA donors upregulate the expression of IL2RB, IL15RA and GZMB. These cells also show upregulate the expression of IL4R and gene inhibitors of the TCR signaling pathway (TIGIT and PD1). On the other hand, TCM CD4+CD69+ cells from DERAA donors upregulate characteristic genes of Th1 cells (STAT1, STAT4), cytotoxic markers (CD161, granzyme A) and crucial genes for TCR signaling (PI3K, AKT, ZAP70, and LAT). TEM and EC CD4+CD69 cells from non-DERAA donors express regulatory transcription factors (FOXP3, STAT3, STAT6, and IRF4). Both subsets also upregulate the expression of IL4R, KLRC3, BCL2, and BCL2A1, potentially contributing to regulatory and antiapoptotic response. Conversely, TEM and EC cells from DERAA donors downregulate IL4R and upregulate CTLA4, TBX21, and genes involved in cytotoxic processes (GZMA, GZMK, PRF1). In DERAA donors, TEM cells express CD27, CD28, PD1 and NF-кB, while EC cells upregulate FAS, BTLA, and IL18R.

Broadly, cells from non-DERAA patients displayed greater expression of chemokine genes. Non-DERAA TN, TCM and TEM cells exhibited heightened expression of CCL20, CCL3, CCL4, CXCL2 and CXCL8 compared to DERAA patients (**Figure 7D**). Regarding cytotoxic molecules, it was notable that TN and TCM cells from non-DERAA displayed increased gene expression of GNLY and GZMB, respectively, compared to DERAA donors. However, CTSW and GZMA genes exhibited significant upregulation across all subsets of cells from DERAA compared to non-DERAA patients. PRF1 and KLRB1 genes also exhibited higher expression in EC and TCM cells from DERAA compared to non-DERAA patients (**Figure 7E**). Inflammatory cytokines characterized cell subsets from non-DERAA patients, including TNF and IFN-γ. However, the expression of inflammatory cytokine receptor genes was increased in DERAA cell subsets, including, IL18R1, IL15RA and IL2RA (**Figure 7F**). In addition, genes related to adhesion, migration, proliferation and differentiation (CD33, ICAM1, LGALS3, THBS1 and FLT3 genes) were DEGs in non-DERAA donors (**Figure 7G**). These findings indicate that cells from DERAA patients, mainly effector CD3+CD4+CD69+ cells display a cytotoxic and proapoptotic expression profile, while cells from non-DERAA are associated with inflammatory and chemotactic genes expression, focusing on survival and anti-apoptotic processes (summarized in **Figure 8**).

## 4 Discussion

Our study has identified peptides that selectively bind to the DERAA-bearing *0103, *0402, *1301, and *1302 HLA-DRB1 alleles, associated with Chagas disease cardiomyopathy (7). Importantly, our findings suggest that the DRB1 allele group may be responsible for the selection of self-derived peptides that share similarities with parasite-derived peptides, potentially contributing to myocardial damage. Molecular mimicry or cross-reactivity, has been observed in Chagas disease (52–55), associated with a persistent inflammatory response against host peptides that structurally or sequentially resemble *T. cruzi*-derived peptides (56–58). Indeed, our results show that activated CD4+ T-cell blasts from CCC but not IND patients become activated and proliferate in the presence of self-peptides that exhibit similarities to parasite-derived peptides. Interestingly, gene expression analysis in the cardiac tissue of CCC patients revealed the upregulation of genes corresponding to the proteins that generate DERAA-selected peptides, such as vimentin and cathepsin S, which are associated with intense fibrotic processes (59, 60) and degradation of the extracellular matrix (61), respectively. Additionally, β2-microglobulin, HLA-DRA, and HLA-DR γ-Chain genes, which were also upregulated, are predominantly expressed in immune cells that are abundant in the intense inflammatory infiltrate observed in the cardiac tissue of CCC patients (62, 63).

We identified immunoglobulin peptides that can selectively bind to DRB1 alleles and exhibit similarity to essential parasite peptides involved in replication, growth, and immune evasion, such as trypanothione (64), trans-sialidase (65), cruzipain (66, 67), and adenylate cyclase receptor (68). Some DERAA associated immunoglobulin peptides were mapped to their variable regions, which act as mirrors of the triggering antigen and have the potential to replace the original peptides in terms of recognition and initiation of an immune response. This concept of idiotypic stimulation (69) has been described in Chagas disease (13, 70). Furthermore, both human and murine models of cancer have demonstrated the existence of idiotype-specific CD4+ T-cell populations that recognize idiotype fragments presented on HLA-II molecules by antigen-presenting cells (71–73). These CD4+ T-cells may be restricted to HLA-DR and do not involve HLA-DP, DQ, or HLA-ABC (73). Therefore, it is conceivable that these immunoglobulins, which share similarities with *T. cruzi*-derived peptides, are also recognized by specific anti-idiotype CD4+ T-cells restricted to DERAA-bearing HLA-DRB1, potentially playing a crucial role in sustaining the immune response under conditions of low parasite load. Dutra et al. (2000) have demonstrated that CD4+ T-cells from CCC and IND can proliferate *in vitro* in response to anti-*T. cruzi* idiotypes isolated from the sera of Chagas disease patients.

Our *in-silico* analysis provides compelling evidence for the significant involvement of the *13 DRB1 allele in the recognition of both self and non-self peptides. HLA genotyping data have consistently demonstrated that the *13 DRB1 allele serves as a protective marker in autoimmune diseases, such as rheumatoid arthritis (74) and type 1 diabetes mellitus (75). However, in infections caused by the *Tropheryma whipplei* bacterium and the *T. cruzi* protozoan, the *13 DRB1 allele has been associated with susceptibility to severe forms of the disease (7, 18). Additionally, our molecular docking analysis revealed that peptides exhibiting high HLA binding affinity also displayed elevated docking score values, providing further support for their potential association with DRB1-DERAA alleles. Overall, the immunogenic self-peptides studied here exhibited the potential to stimulate the production of IL-4 or both IL-4 and IFN-γ. In mice, while IL-4 plays a critical role in controlling myocarditis and inhibiting Th1 cell responses that produce IFN-γ (76), it is also implicated in intracellular parasite replication (77) and, notably, in the phosphorylation of STAT6 in cardiac fibroblasts, leading to increased collagen production (78). This process contributes to cardiac fibrotic remodeling and dysfunction (79) observed in patients with CCC (12, 80–82).

To characterize the involvement of self and non-self peptides in the context of Chagas disease, we investigated whether these peptides could elicit an immune response in patients with distinct clinical forms of the disease. Our findings revealed a notable difference in the proliferative response of activated CD4+ T cell blasts to immunoglobulins 1 and 2 and cathepsin S peptides, which share amino acid similarity with adenylate cyclase receptor, trypanothione, cruzipain, and trans-sialidase, respectively. This enhanced proliferation was exclusively observed in CCC patients, highlighting the potential role of cross-reactivity phenomena in the progression of Chagas cardiomyopathy (52, 83–85). Remarkably, it has been demonstrated that CD4+ T-cells from Chagas disease patients possess the ability to proliferate when exposed to the P214-cruzipan epitope, which exhibits structural similarity to the catalytic domain of cathepsin S (86). Hence, it is conceivable that T-cells can recognize parasite-derived peptides, and we have presented compelling evidence that ACR, trans-sialidase, and trypanothione peptides selectively activate CD4+ T-cell blasts in patients with CCC, while remaining inactive in IND. These parasite-derived peptides are capable of activating diverse subsets of CD4+ T-cells, particularly IFN-γ+CD4+ T-cells in severe cases of Chagas disease, upon stimulation by members of the trans-sialidase superfamily (87). Proliferating index was positively correlated with production of IL-4, CXCL9 and CCL11, potentially involved in fibrosis and recruitment of inflammatory cells. CXCL9 has been associated with Chagas cardiomyopathy (88). We observed a similar response of CD4+ T cells from patients with and without DERAA to parasite-derived peptides but, interestingly a higher response from CD4+ T cells from DERAA+ patients to Ig1, MBP and CF VIII. Of these, response to MBP seemed to be the strongest. MBP is associated with the autoimmune disease multiple sclerosis, being one of the key autoantigens implicated in the disease. The significance of the response of CD4+ T cells from Chagas patients that display DERAA to MBP is unclear, but it is tempting to hypothesize that this response might be associated with auto-reactivity in Chagas disease. It is noteworthy to mention that heart dysfunction in Chagas disease often includes nervous conductive alterations (51).

HLA typing was performed on all donors included in our study, revealing that the HLA-DRB1 allele was expressed by 40% of CCC patients, compared to a frequency of 20% among IND. The impact of HLA-II polymorphism on the progression of Chagas disease has been the focus of investigations. While a lower prevalence of DRB1*0301, *0303, *14 (89, 90), DPB1*0101 (91), and DQB1*06 (92) alleles has been suggested to confer protection against chronic infection, a higher frequency of DRB1*0103, *0402, *08, *1301, 1302, 1503 (7, 91, 93), DQB1*0501 (89), and DPB1*0401 (91) alleles has been associated with the recognition of antigens that can trigger cardiac damage, arrhythmia, and congestive heart failure in chronic Chagas disease. These findings emphasize the potential involvement of HLA-II gene alleles as determinants in the development of different clinical forms of Chagas disease.

The activation of CD4+ T cell blasts induced by immunoglobulin 1, cathepsin S, and all parasite-derived peptides exhibited a negative association with LVEF and a positive correlation with LVSD. Decreased LVEF and an increased frequency of CCR5+ T-cells are indicative of an unfavorable prognosis, reflecting the cardiac remodeling observed in CCC (94). Similar patterns have been observed in other cardiac pathologies, such as viral myocarditis and idiopathic dilated cardiomyopathy, where pathogens have been implicated in the generation of autoreactive T-cells, promoting cross-reactivity in genetically susceptible individuals (95). Additionally, the presence of perforin-expressing CD4+ cytotoxic T-cells has shown an inverse relationship with LVEF (96), while an increase in Foxp3+high CD25+high CD4+ T-cells and IL-17 expression has been associated with a positive LVEF and improved cardiac function in human Chagas disease (97, 98). Furthermore, our findings revealed a correlation between IL-4 and activated CD4+ T cell blasts stimulated by self and non-self peptides in CCC. Considering that LVEF and LVSD are important markers of Chagas cardiomyopathy severity and predictors or mortality (51), the recognition of self-peptides further supports the pathogenic role of cross-reactivity in CCC. An important factor contributing to autoreactivity in Chagas disease may be the release of parasite-derived material during the lytic cycle of *Trypanosoma cruzi*. As infected host cells rupture, both parasite antigens and host-derived molecular components are exposed to the immune system, potentially triggering bystander activation and epitope spreading. This process could lead to the recognition of self-antigens in a molecular mimicry-independent manner, reinforcing the chronic immune activation observed in Chagas cardiomyopathy (52, 53, 55, 57). While our study primarily focused on HLA-associated peptide presentation, the role of parasite-induced cell lysis in amplifying autoreactivity represents an additional mechanism worthy of further investigation.

Single-cell RNA sequencing analysis of CD4+CD69+ T-cells from DERAA and non-DERAA patients demonstrated marked differences in the two groups through the analysis of 400 immunological genes, in which cells from DERAA patients display a highly cytolytic profile, with augmented expression of transcripts that code for proapoptotic proteins and molecules involved in cytotoxic processes, while non-DERAA donor cells demonstrated high expression of genes that coordinate inflammatory, chemotactic, migratory and differentiation responses. These same characteristics were observed when cells were further subdivided in memory cell subpopulations. Thus, activation of these T-cells may be critical in mediating disease pathology by orchestrating the immune response and perhaps by directly mediating tissue destruction. In favor of this hypothesis, the role of CD4+ cytotoxic T-cells has been shown in the murine model of Chagas disease (96), and has been markedly associated with human Chagas cardiomyopathy (99–101).

As limitations of the study, we employed a small number of Chagas patients. This is related to the importance of identifying a clinically well-characterized group of patients to perform these costly and detailed analysis. These patients, which have been followed at the UFMG hospital for over 20 years, were carefully selected to be in the polar spectra of Chagas disease clinical forms, unequivocally classified as IND or CCC. In addition, the single cell analysis method employed used a targeted panel of 389 genes, and not a full RNA sequencing platform. While this panel was chosen for containing immune-response genes, a full sequencing analysis could provide additional information. Through our integrated *in silico* and *in vitro* approach, we have successfully identified potential antigenic targets capable of modulating the immune response in individuals expressing specific HLA-DRB1 alleles bearing the DERAA motif. These targets carry significant implications for influencing the clinical outcome of Chagas disease. Although our peptide selection was based on DERAA and not all peptides elicited significant responses from different T-cell subsets, the interaction between the ligand and HLA-II molecules is crucial for immunogenicity, albeit not the sole factor in inducing T-cell responses. Nevertheless, predictive tools assist in identifying the most promising antigenic targets that have the potential for modulation, as demonstrated by the response to immunoglobulins, cathepsin S, and all parasite-derived peptides. Importantly, our findings serve as a foundation for the development of strategies aimed at counteracting the activity of these peptides, ultimately impeding disease progression.

## Data Availability

Data have been deposited in the Gene Expression Omnibus (GEO) under accession number GSE295194.
