# Supplementary material for: Self and parasite-derived peptides selected upon DERAA-bearing HLA-DRB1 alleles activate CD4+ T cells from Chagas cardiomyopathy patients and are associated with ventricular dysfunction
Source: Front Immunol. 2025 May 5;16:1527115. doi: 10.3389/fimmu.2025.1527115 (PMC12086171; doi:10.3389/fimmu.2025.1527115)
Supplement: Supplementary file 1 [file Table1.docx]

Supplementary Material

Self and parasite-derived peptides selected upon DERAA-bearing HLA-DRB1 alleles activate CD4+ T cells from Chagas cardiomyopathy patients and are associated with ventricular dysfunction

*** Correspondence:**Correspondent author: Walderez Ornelas Dutra*
waldutra@gmail.com

**Supplementary Table 1**. Pilot assay demonstrating low proliferating index of *in vitro* culture lymphocytes.

| **Stimulus** | **%Lymphocytes** | **%CD4+CD69+ T cells** | **%CD8+CD69+ T cells** | **%CD19+HLA+ cells** |
| --- | --- | --- | --- | --- |
| Medium+anti-CD28 | 4.79 | 0 | 0 | 0 |
| Trypanothione | 1.82 | 0 | 0.34 | 0 |
| Trans-sialidase | 1.55 | 1.33 | 0 | 0 |
| Immunoglobulin 1 | 3.41 | 0 | 0 | 0 |
| Immunoglobulin 2 | 1.66 | 1.78 | 0 | 0 |
| Cathepsin S | 2.71 | 0.57 | 0 | 0 |
| Adenylate cyclase receptor | 2.43 | 1.61 | 8.25 | 0 |
| Vimentin | 1.41 | 5.88 | 3.7 | 0 |
| Myelin Basic protein | 2.8 | 3.06 | 5.95 | 0 |
| Coagulation factor VIII | 6.41 | 2.61 | 6.71 | 0 |
| Cruzipain | 3.02 | 2.98 | 2.85 | 0 |

**Supplementary table 2.** Prediction of human-derived and *Trypanosoma cruzi-*derived peptides-HLA-DRB1 binding affinity.

| **Protein** | **Peptide Sequence** | Length | Region | **Alleles of HLA-DRB1** | | | | | | | |
| --- | --- | --- | --- | --- | --- | --- | --- | --- | --- | --- | --- |
|  |  |  |  | Allele *0103 | | Allele *0402 | | Allele *1301 | | Allele *1302 | |
|  |  |  |  | nM  (Aff) | Rank (%) | nM (Aff) | Rank (%) | nM (Aff) | Rank (%) | nM  (Aff) | Rank (%) |
| Vimentin | SAVRLRSSVPGVR | 13 | 66-78 | 4563 | 36 | 2073 | 17 | 1035 | 31 | 1953 | 39 |
| Enolase-alpha | IFDSRGNPTVEVDLF | 15 | 11-25 | 5145 | 41 | 9646 | 71 | 3411 | 57 | 1032 | 26 |
| Myelin Basic Protein | VHFFKNIVTPRTP | 13 | 114-126 | 2153 | 16 | 1498 | 11 | 1107 | 32 | 556^+^ | 16 |
|  | KIFKLGGRDSRS | 12 | 178-190 | 6341 | 49 | 7221 | 59 | 3638 | 58 | 8677 | 77 |
| β2 Microglobulin | TPKIQVYSRHPAEN | 14 | 24-37 | 2794 | 22 | 1047 | 6 | 219^+^ | 9 | 1654 | 35 |
| HLA-DR α-Chain | LANIAVDKANLEI | 13 | 85-97 | 9736 | 68 | 5593 | 48 | 3127 | 55 | 871^+^ | 23 |
|  | IIKGLRKSNAAERRG | 15 | 237-251 | 1435 | 10 | 809^+^ | 4 | 37^++^ | 0.8 | 281^+^ | 8 |
| Cathepsin S | DPTLDHHWHLWKKTYGKQYK | 20 | 21-40 | 3305 | 26 | 35^++^ | 31 | 668^+^ | 23 | 2789 | 48 |
| COL2A1 | PGIAGFKGEQG | 11 | 458-468 | 18591 | 94 | 10455 | 74 | 18132 | 95 | 23720 | 95 |
|  | LQYMRADQAAGGLR | 14 | 1235-1249 | 3249 | 25 | 2566 | 22 | 2174 | 46 | 1243 | 29 |
| Immunoglobulins | KVQWKVDNALQSG | 13 | 37-50 | 3415 | 27 | 2647 | 23 | 1715 | 41 | 498^+^ | 15 |
|  | IQVSWLREGKQVGSG | 15 | 144-158 | 4369 | 35 | 2988 | 26 | 497^+^ | 18 | 2583 | 46 |
|  | ASGGTFSSFAINWVRQAPGQ | 20 | 24-43 | 588^+^ | 3 | 612^+^ | 3 | 169^+^ | 7 | 64^++^ | 2 |
|  | SKNTVYLQIDSLRAEDTA | 18 | 75-92 | 1664 | 12 | 1665 | 13 | 1836 | 42 | 2122 | 41 |
|  | KPGQPPRLLIYDASNRATGIPA | 22 | 22-43 | 1363 | 9 | 674^+^ | 3 | 172^+^ | 7 | 56^++^ | 2 |
| Coagulation Factor VIII | EPRKNFVKPNETKTYFWKVQ | 20 | 1819-1839 | 5312 | 42 | 5853 | 50 | 411^+^ | 16 | 158^+^ | 5 |
|  | EVGDTLLIIFKNQASRPYNI | 20 | 475-495 | 403^+^ | 2 | 88^++^ | 0.02 | 33^++^ | 0.6 | 33^++^ | 0.7 |
| Calpain | SGVTFGSALSQLTGGITFAIDW | 22 | 344-366 | 2485 | 18 | 5454 | 47 | 1812 | 42 | 2298 | 43 |
| Trans-sialidase | WKDLDNALQS | 10 | 89-99 | 26572 | 95 | 26403 | 95 | 24193 | 95 | 20764 | 95 |
|  | PAGASEEGSRGD | 12 | 906-918 | 31108 | 95 | 33772 | 95 | 33600 | 95 | 3780 | 95 |
| Adenylate cyclase receptor | TVYIKKFVESFRAEDVA | 17 | 498-515 | 1424 | 10 | 1335 | 9 | 257^+^ | 11 | 788^+^ | 21 |
| CDC16 | VYQIDPFRAE | 10 | 448-458 | 16622 | 90 | 17853 | 92 | 12930 | 88 | 1233 | 86 |
| Rab1 small GTP-binding protein | RLLQTAGIIEDASSRTTGGWIP | 22 | 32-55 | 7515 | 57 | 7317 | 59 | 2957 | 53 | 1313 | 30 |
| Enolase | ILDSRGNPTVEVEV | 14 | 10-24 | 9989 | 69 | 12635 | 81 | 7396 | 76 | 2522 | 45 |
| Cruzipain | EETLTSQFAEFKQKHGRVYE | 20 | 30-50 | 1958 | 15 | 2862 | 25 | 495^+^ | 18 | 1573 | 34 |
| GPI-PLC | VYLNILRAE | 9 | 356-364 | 24088 | 95 | 22199 | 95 | 12069 | 87 | 6956 | 72 |
| Anion-transporting ATPase | SKYLAQIDSL | 10 | 302-311 | 25115 | 95 | 25675 | 95 | 21071 | 95 | 2112 | 95 |
| Retrotransposon Hot spot protein | QINSLRLAEE | 10 | 122-131 | 25474 | 95 | 21816 | 95 | 18833 | 95 | 2238 | 95 |
| Ins(1,4,5)P3 | RLLIHEASSR | 10 | 134-143 | 14069 | 84 | 10538 | 74 | 2156 | 46 | 6062 | 68 |
| Trypanothione | GHPLLIYNVASR | 12 | 24-35 | 9358 | 67 | 3377 | 29 | 1294 | 35 | 3396 | 53 |
| Reverse transcriptase SLAC | VSWREGKQRVLTEEL | 15 | 266-282 | 3979 | 32 | 8648 | 66 | 1422 | 36 | 4518 | 61 |

**+**Indicate intermediate binding affinity peptide; ++Indicate strong binding affinity peptide; nM(Aff): binding affinity in nanomolar. GPI-PLC: Glycosylphosphatidylinositol-specific phospholipase C; Ins(1,4,5)P3: Inositol-1,4,5-trisphosphate (IP3) 5-phosphatase.

**Supplementary table 3.** Docking score of peptides with intermediate or strong binding affinity to HLA-DRB1.

| **Protein** | **Peptide** | **nM (Aff)** | **Docking Score** |
| --- | --- | --- | --- |

| Myelin Basic Protein | VHFFKNIVTPRTP | 556 (*1302) | -234.242 |
| --- | --- | --- | --- |
| β2 Microglobulin | TPKIQVYSRHPAEN | 218 (*1301) | -229.674 |
| HLA-DR-α Chain | LANIAVDKANLEI | 871 (*1302) | -190.873 |
|  | IIKGLRKSNAAERRG | 809 (*0402) 37 (*1301) 281 (*1302) | -187.426  -194.818  -194.819 |
| Cathepsin S | DPTLDHHWHLWKKTYGKQYK | 35 (*0402) 668 (*1301) | -243.831  -268.104 |
| Immunoglobulin | KVQWKVDNALQSG | 498 (*1302) | -241.092 |
|  | IQVSWLREGKQVGSG | 497 (*1301) | -214.437 |
|  | ASGGTFSSFAINWVRQAPGQ | 588 (*0103)  612 (*0402)  169 (*1301)  64 (*1302) | -264.328  -246.379  -255.617  -255.617 |
|  | KPGQPPRLLIYDASNRATGIPA | 674 (*0402) 172 (*1301)  56 (*1302) | -231.386  -246.430  -246.430 |
| Coagulation Factor VIII | EPRKNFVKPNETKTYFWKVQ | 411 (*1301)  158 (*1302) | -236.128 -236.128 |
|  | EVGDTLLIIFKNQASRPYNI | 403 (*0103)  88 (*0402)  33 (*1301)  33 (*1302) | -239.805  -249.993  -239.453  -239.453 |
| Adenylate cyclase receptor | TVYIKKFVESFRAEDVA | 257 (*1301) 788 (*1302) | -213.749 -213.749 |
| Cruzipain | EETLTSQFAEFKQKHGRVYE | 495 (*1301) | -233.832 |

nM (Aff): Binding affinity in nanomolar.

**Supplementary Table 4**. Alignment of myelin basic protein peptide (VHFFKNIVTPRTP) conserved in distinct species.

| **Specie/region** | **Sequency** |
| --- | --- |
| **Human/220-226** | **V V H F F K N** IVTPRTP |
| Bovin/85-91 | **V V H F F K N** |
| Carob/49-55 | **V V H F F K N** |
| Cavpo/85-91 | **V V H F F K N** |
| Chick/85-91 | **V V H F F K N** |
| Hetfr/75-81 | **V V H F F K N** |
| Leuer/75-81 | **V V H F F K N** |
| Mouse/217-223 | **V V H F F K N** |
| Pantr/86-92 | **V V H F F K N** |
| Pig/86-92 | **V V H F F K N** |
| Rabit/84-90 | **V V H F F K N** |
| Rat/110-116 | **V V H F F K N** |
| Squac/75-81 | **V V H F F K N** |

*Green color represents the region from human MBP, compared to other species.

**
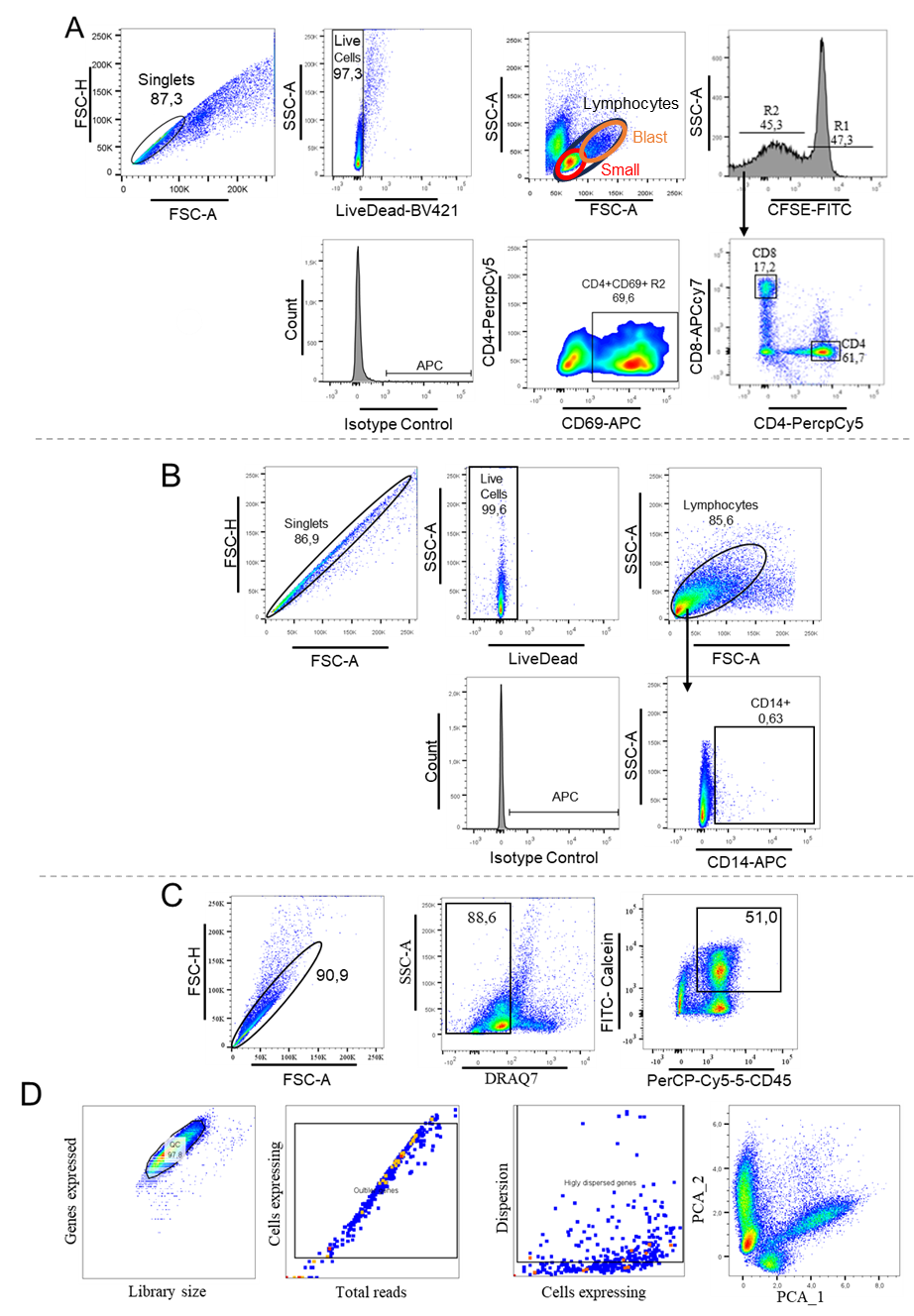
Supplementary Figure 1.**

Supplementary Figure 1: Representative figure of the gated strategy used in the study. (A) Parameters FSC-A x FSC-H were used to remove doublets; live cells were selected according to SSC-A and Live-Dead-BV421. Total lymphocytes, small and blast cells were selected according to size (FSC-A) and granularity (SSC-A) parameters. For separation of non-proliferating cells (R1) and proliferating (R2), CFSE was used. For phenotypic separation of lymphocyte subpopulations, CD4, CD8 and CD19 surface markers were selected, followed by selection of CD69 to identify CD4+CD69+ and CD8+CD69+ lymphocytes, whereas HLA-DR was selected to identify CD19+HLA-DR+ lymphocytes. (B) The analysis strategy used to demonstrate absence of CD14+ cells after 120h of stimuli. Parameter SSC-A and CD14-APC marker in total lymphocytes was selected. (C) For scRNAseq, sorted leukocytes were stained with CD45 (PercpCy5.5), DRAQ7 (APC-H7) and calcein (FITC) antibodies. Parameters FSC-A x FSC-H were used to remove doublets; live cells were selected according to Calcein-FITC and CD45-PerCP-Cy5-5. (D) Data of single-cells were evaluated considering quality cells (QC), log normalization and Outliers genes, as demonstrated in plots.


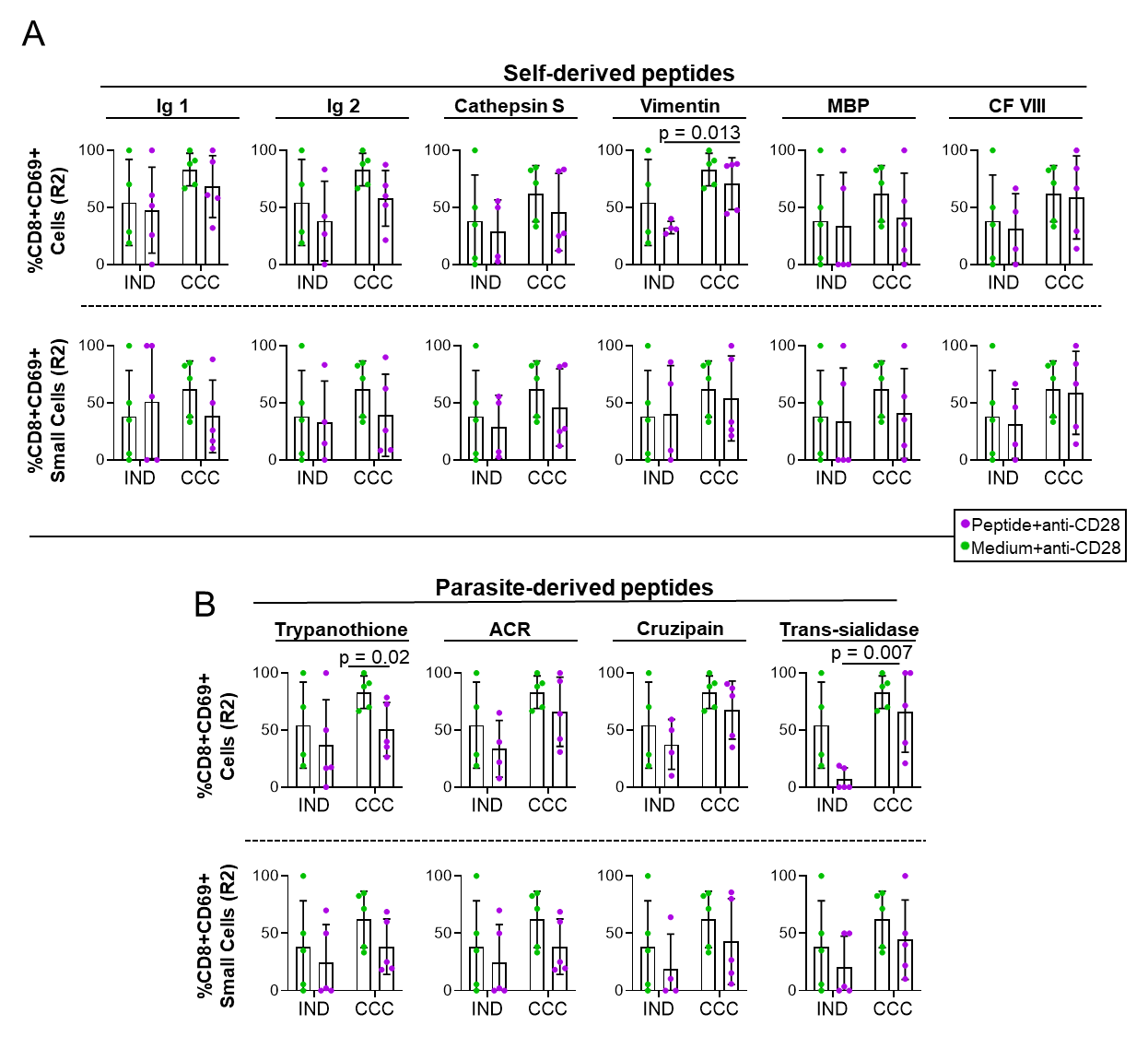
**Supplementary Figure 2**

Supplementary Figure 2. Frequency of activated CD8+CD69+ total lymphocytes and small cells after stimulation with self-derived and *T. cruzi*-derived peptides. (A) Frequency (%) of CD4+CD69+ T lymphocytes and small cells proliferating (R2) from indeterminate (IND, n=5) and chronic Chagas cardiomyopathy (CCC, n=5) patients stimulated with medium and anti-CD28 or self-peptides. (B) Frequency (%) of CD8+CD69+ T lymphocytes and small cells proliferating (R2) from IND and CCC patients stimulated with medium and anti-CD28 or parasite-derived peptide. The analysis was performed as described in the materials and methods for comparison between the groups. Significant differences with p < 0.05 are demonstrated.

**
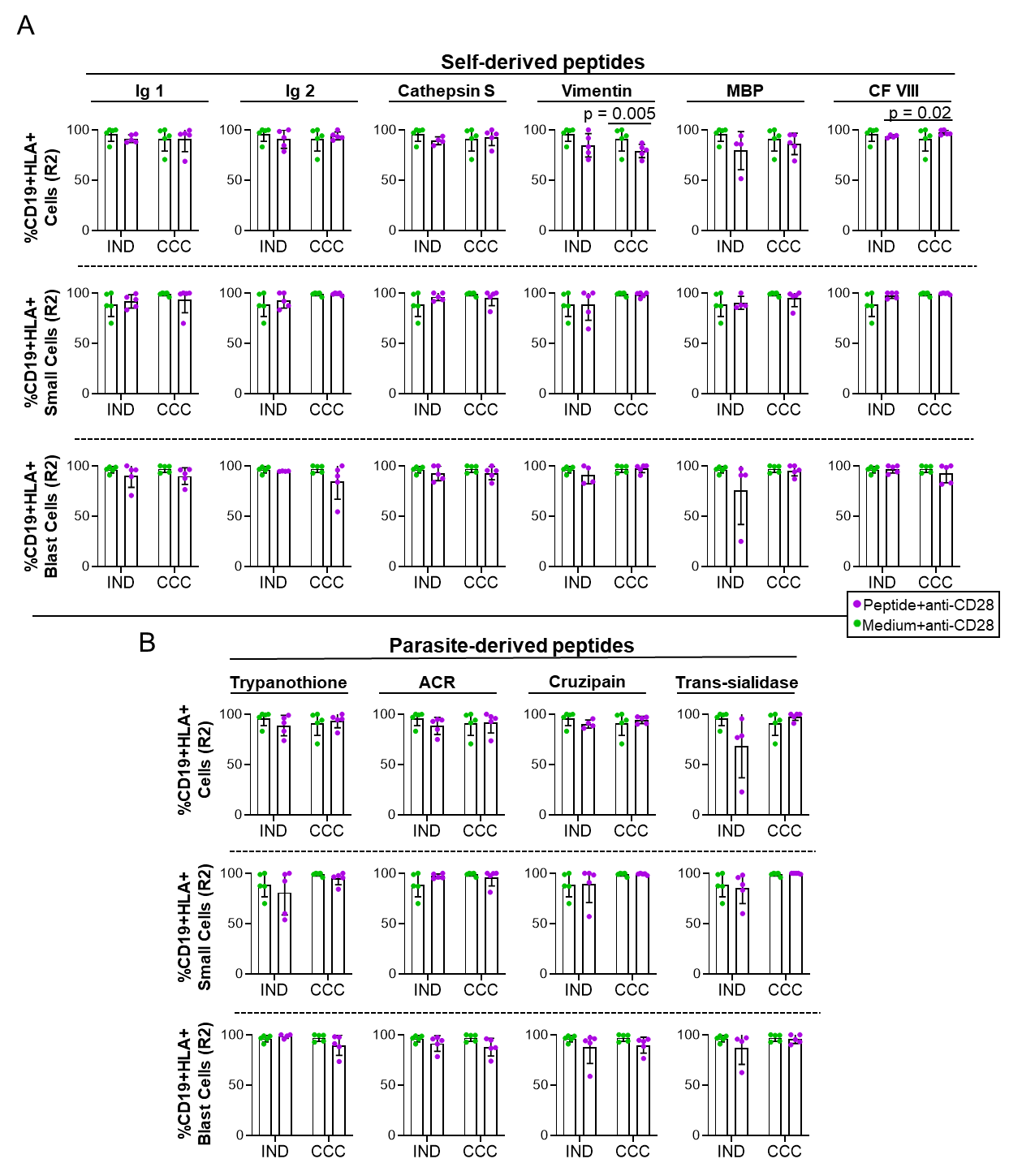
Supplementary Figure 3**

Supplementary Figure 3. Frequency of CD19+HLA-DR+ total lymphocytes, small and blast cells after stimulation with self-derived and *T. cruzi*-derived peptides. (A) Frequency (%) of CD19+HLA-DR+ T lymphocytes, small and blast cells proliferating (R2) from indeterminate (IND, n=5) and chronic Chagas cardiomyopathy (CCC, n=5) patients stimulated with medium and anti-CD28 or self-peptide. (B) Frequency (%) of CD19+HLA-DR+ T lymphocytes, small and blast cells proliferating (R2) from IND and CCC patients stimulated with medium and anti-CD28 or parasite-derived peptide. The analysis was performed as described in the materials and methods for comparison between the groups. Significant differences with p < 0.05 are demonstrated.

**Supplementary Figure 4**

Supplementary Figure 4. Plasma levels of soluble molecules in different clinical forms of Chagas disease. Plasma levels of (A) cytokines, (B) cytokine receptors, (C) chemokines and (D) growth factors measured using Bio-Plex Pro TM Human Cytokine Standard platform, as described in material and methods, were analyzed in patients with indeterminate (IND, n=5, green dots) and chronic Chagas cardiomyopathy (CCC, n=5, purple dots) clinical forms of Chagas disease. The results are shown as mean values (pg/mL).
